# The experience and supportive care needs in people affected by ovarian cancer and their informal caregivers: a qualitative systematic review

**DOI:** 10.1007/s00520-026-10542-z

**Published:** 2026-03-23

**Authors:** J. Davey, A. Collier, M. Turner, C. Paterson

**Affiliations:** 1 Flinders University, Caring Future Institute, Adelaide, Australia; 2 Central Adelaide Local Health Network, Adelaide, Australia; 3 University of Canberra, Canberra, Australia; 4 Southern Adelaide Local Health Network (SALHN) Flinders Medical Centre, Adelaide, Australia; 5 Research Centre for Palliative Care Death and Dying, Flinders University, Adelaide, Australia

**Keywords:** Systematic review, Ovarian cancer, Supportive care, Patients, Informal caregivers

## Abstract

**Purpose:**

To critically synthesise qualitative research to understand the experiences of supportive care needs in people affected by ovarian cancer and their informal caregivers.

**Method:**

A qualitative systematic review has been reported according to the Preferred Reporting Items for Systematic Reviews and Meta-Analysis (PRISMA) guidelines. The Joanna Briggs meta-aggregation methodology was utilised. Electronic databases were searched for all qualitative studies irrespective of research design by an expert systematic review librarian. Data extraction and methodological quality assessment were performed.

**Results:**

A total of 26 studies were included which represented a total sample of 962 participants inclusive of 842 patients and 120 informal caregivers. There were a total of 133 individual findings included in this review, which were synthesised into four main findings that emerged: (1) awareness around ovarian cancer, (2) communication in the healthcare sector, (3) everything that comes with the disease, (4) what the future holds.

**Conclusion:**

This review identified that while some women living with ovarian cancer experienced suffering and distress, others reported emotional wellbeing and comfort needs met. There were gaps in service from both the informal caregiver and women ranging from before diagnosis even to post-treatment and beyond. Service redesign needs to occur with a focus on (1) improved awareness around ovarian cancer signs and symptoms, (2) effective communication strategies within and across healthcare providers, (3) increased information and support for both women and their informal caregiver throughout the cancer trajectory, and (4) developing survivorship care plans to promote wellness.

**Supplementary Information:**

The online version contains supplementary material available at 10.1007/s00520-026-10542-z.

## Introduction

Over 300,000 women globally are diagnosed with ovarian cancer every year [1]. Ovarian cancer is commonly diagnosed after the age of menopause but can affect women of all ages [2]. Moreover, awareness about the early signs and symptoms of ovarian cancer can be suboptimal in healthcare settings and public health initiatives [3]. Recent evidence [4] has indicated that 69% of newly diagnosed women did not know about the presenting signs and symptoms of ovarian cancer [5].

Despite the growing number of people affected by ovarian cancer, there has been little improvement in survival rates, often because women present with advanced stage disease due to late presentations [6]. The vagueness of symptoms at presentation and the lack of effective strategies for routine screening mean that ovarian cancer often goes undiagnosed and/or diagnosed at an advanced stage. Thus, advanced cancer stage diagnosis means that curative treatment is not possible or is very limited to a small proportion of women [6]. Treatments often involve chemotherapy, radiotherapy and surgery, resulting in long-term negative health impacts reducing quality of life [7]. Supportive care is defined as necessary services for those living with or affected by cancer [8]. Supportive care addresses individuals’ physical, psychological, spiritual, and social needs during diagnosis, treatment, or follow-up phases of the cancer care continuum [8]. Supportive care provisions are modifiable factors that healthcare providers can change to enhance safe, equitable, timely, and accessible care for people diagnosed with ovarian cancer and their families and adjunct support networks.

Many people with ovarian cancer require multiple supportive care services to improve multidimensional aspects of wellbeing. At the same time, evidence underscores that access to supportive care remains unco-ordinated and fragmented across primary and tertiary care settings, and consequently the care burden indirectly falls to informal (unpaid) caregivers [8]. Further, cancer healthcare professionals continue to report challenges to meeting supportive care needs due to the well-documented workforce shortages, burnout, and increased demands for cancer services due to an ageing population [6]. Evidence has identified a causal relationship between the frequency of unmet supportive care needs and reduced quality of life in people affected by cancer [8].

Specific to the gynaecology oncology population group, evidence data has also identified that unmet supportive care needs are associated with reduced psychosocial outcomes [9]. Emerging evidence [10] highlights that people affected by ovarian cancer experience a range of unmet supportive care needs, despite routine clinical follow-up, and this is further influenced by geographical location. For example, people living in rural and remote areas generally find travel and accommodation to the hospital for treatment and clinical review with healthcare professionals difficult, and this is often compounded by financial toxicity [10]. Research shows that caregiver distress can have an impact on patients’ distress, as well as long-term anxiety and coping adjustments [11]. Therefore, addressing both patients and caregivers’ unmet supportive care needs is central to improving overall care for those affected by ovarian cancer and the treatment trajectory. To date, there has not been a systematic review of the empirical literature to critically synthesise the supportive care needs of women diagnosed with ovarian cancer and their families. This critical lack of evidence synthesis is an important omission given the lack of health promotion on early signs and symptoms of this disease to people living with advanced cancer at diagnosis, in comparison to other cancer populations. Therefore, this qualitative systematic review aimed to identify the supportive care needs of women diagnosed with ovarian cancer and their informal caregivers.

The qualitative systematic review addressed the following research questions:

1. What are the unmet supportive care needs among people affected by ovarian cancer and their informal caregivers?
2. What types of supports were perceived as beneficial among people affected by ovarian cancer and their informal caregivers?

## Methods

### Design

A qualitative systematic review was completed in accordance with Joanna Briggs Institute (JBI) meta-aggregation methodology [12] and a priori research protocol registered with PROSPERO (CRD420251115817). A systematic review provides reliable, unbiased summaries of findings which can inform evidence-based decisions and future policies among people affected by ovarian cancer [13]. Qualitative research provides insight to complex topics, understanding the “why” behind human behaviour; therefore, this methodology was chosen as the sole criteria for this systematic review. The JBI methodology follows a systematic and structured framework [12]. This systematic review has been reported according to the Preferred Reporting Items for Systematic Reviews and Meta-analyses (PRISMA) [14]. Supplementary file 1 includes the completed PRISMA checklist.

### Searches

The CINAHL, Medline, PsycINFO, Scopus, Web of Science Core Collection databases, and Google Scholar were searched for the timeframe of August 2015 to August 2025. This timeframe was chosen as a team to ensure the inclusion of journal articles relevant to contemporary healthcare and changes in the treatments of ovarian cancer. The search architecture was developed in consultation with a highly experienced research librarian (MT) and was based on the efficient and complete method of searching developed at Erasmus University Medical Centre [13]. The combination of keywords and relevant subject headings was based on the broad concepts of “Sample”—people affected by ovarian cancer, “Phenomenon of Interest”—experiences, needs, and preferences for supportive care, and “Research Type”—qualitative studies. A limiter was applied to the database searches for studies published in English language. Additionally, the reference lists of all included articles were searched to identify any further potentially relevant studies. Endnote software was utilised for citation management. See Supplementary file 2 for the full record of database searches.

### Study eligibility criteria and screening

Following the searches, all identified records were imported into Covidence systematic review software for de-duplication and the study selection process. Titles and abstracts were screened according to the inclusion and exclusion criteria by three reviewers (JD, CP, MT); any conflicts were resolved by one reviewer (AC). All full-text studies that met the criteria were then assessed in detail by two reviewers (JD, CP). Any conflicts were then reviewed again by one reviewer (AC). Excluded studies at full-text review are listed with reasons in Supplementary file 3.

### Population

#### Inclusion

- People over 18 years with a diagnosis of ovarian cancer and their informal caregivers.

#### Exclusion

- People under 18 years and that do not have a diagnosis of ovarian cancer.

### Intervention/exposure

#### Inclusion

- Studies included were those exploring experiences, needs, and preferences for supportive care in participants diagnosed with ovarian cancer and their informal caregivers.
- Relevant systematic reviews were scrutinised for potentially relevant studies.
- Study period (August 2015–August 2025) with the rationale due to the significant change in the clinical management landscape of people affected by ovarian cancer, i.e. Breast Cancer gene (BRACA) testing for family links and poly-ADP ribose polymerase (PARPi) approved 2015 [15].

### Exclusion

Studies conducted with patients with mixed cancer groups, except when separate sub-group analyses of only ovarian cancer patients were reported. Commentaries, editorials, reviews, and studies that did not report on experiences, needs, and preferences for supportive care were not explicitly reported.

### Study characteristics

#### Inclusion

All types of qualitative research designs irrespective of methodology.

### Exclusion

- All types of quantitative and mixed methods research designs.
- Editorials, commentaries, conference posters, conference abstracts.

### Data extraction

Information about the population group, context, geographical location, and study methods were extracted. Findings were extracted by (JD) from the included studies according to guidance provided by the JBI meta-aggregation method; findings and illustrative quotations were used to create supporting categories and quality checked with reviewers (CP, AC) [12]. Data extraction was piloted on a small group of five studies and refined by consensus among the research team and then proceeded to full data extraction for all included studies. The data extraction was organised using Microsoft Word. The data extraction (findings and illustrative quotes) from the main findings of the original studies were extracted in tabular format.

### Assessment of methodological quality

The JBI Critical Appraisal Checklist for Qualitative Research was used to assess all studies that met the inclusion criteria. The checklist is a 10-item assessment tool that evaluates the congruity between study methodology, the research question, interpretation of the findings, philosophical/theoretical position adopted in the study, and the representation of the data [12]. Yes, no, or unclear was the rating system used to assign scores to each research paper.

### Data synthesis

Reflexive thematic analysis was used which involved identifying, analysing, and reporting patterns and differences within the data extracted [16]. Meta-aggregation analysis was used to extract findings and synthesise data from multiple studies which was achieved by following a sequential and dynamic three-step process [12]. Step one involved immersive reading and re-reading of all the findings. Step two comprised generating initial categories of similar and divergent candidate themes, continually checking coherence and relevance across the entire dataset. Step three involved refining the overall synthesised findings and weaving data extracts to provide an analytical narrative [12]. Throughout the data synthesis process, weekly meetings were held with all researchers to ensure consensus and rigour.

Each finding was assessed and given a ConQual ranking of either “unequivocal” (clear association between the finding and illustration), “credible” (unclear association between the finding and illustration, leaving it open to challenge), or “not supported” (findings not supported by data) [12]. An illustration is a direct quotation from the paper to support the authors’ findings [16]. Unsupported findings were not included in the final synthesis in keeping with the JBI methodology [12].

## Results

**Fig. 1:**
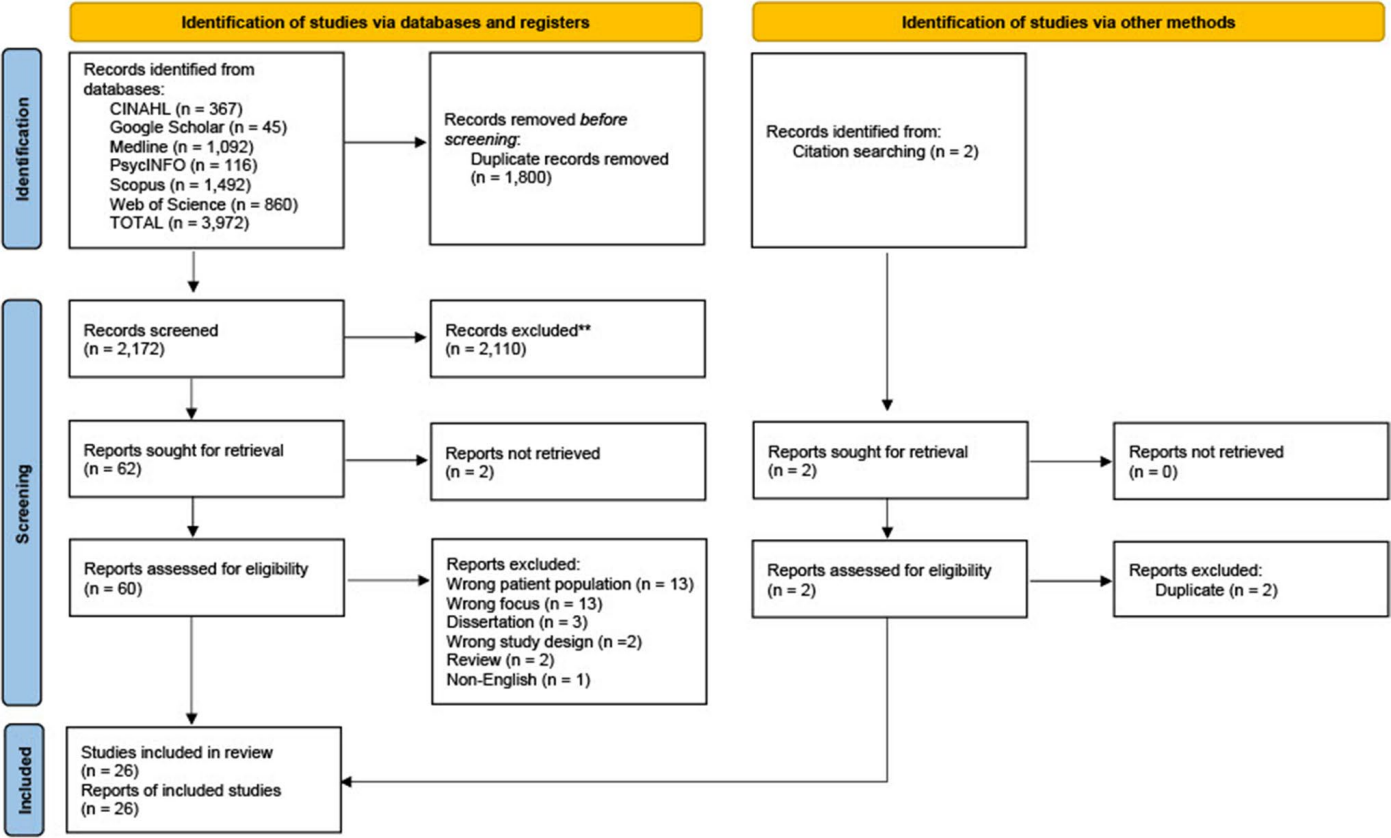
PRISMA flow diagram

Following the removal of duplicates, a total of 2172 publications were screened by title and abstract. After the initial screening, 60 full-text reports were assessed according to eligibility criteria. Thirty-four of these were excluded with reasons included as seen in Supplementary file 3. A PRISMA flow diagram is provided to show the study selection process [14] (Fig. 1). Twenty-six met the inclusion criteria. Studies were conducted in a range of countries, including the UK (*n* = 2), the USA (*n* = 11), Canada (*n* = 2), Taiwan (*n* = 2), Malaysia (*n* = 1), Japan (*n* = 1), Poland (*n* = 1), and Australia (*n* = 6). Table 1 includes an overview and further details of the included studies. The total representation of participants in the included studies was 962; of these, 842 were patients and 120 informal caregivers. The overall quality of the included studies was moderate to good; however, commonly, there was no mention of the researcher reflexivity and theoretical framework adopted. There were limited studies that detailed the transparency of how the researcher’s culture, healthcare discipline, epistemological, and ontological positioning influenced the research and vice versa; see Table 2.

**Table 1:** Characteristic of all systematic review included publication

Study and country	Phenomena of interest	Qualitative methods for data collection and analysis theoretical model	Setting/context/culture	Participant characteristics and sample size	Description of main findings
Arida et al. (2019) USA Pennsylvania—Pittsburgh	Experience of mothers with ovarian cancer, including the interaction between their roles as mothers and patients with cancer	Method: secondary analysis of focus groups Analysis: descriptive coding using terms “mom” etc. Theoretical model: adaptive framework analysis Methodology: not stated	Focus groups recruited from randomised clinical trial. Four audio recording. All held at local café	No. 13 patients Gender: Not stated Ethnicity: 100% white Age: 100% > 18 years Treatments: 50% currently receiving treatment	The overarching theme described by the women in this study was that of role transformation. Women described this transformative process as one in which they took on specific role, first of being someone receiving the diagnosis of ovarian cancer, then taking the identity of a patient with cancer, and finally as someone who is simultaneously and inextricably a mother and a patient with cancer
Boba et al. (2021) AUS Perth, WA	To explore and identify their view of the health symptoms and outcomes that matter most to them as they traverse their disease pathway	Method: community conversation, interview, and focus group Analysis: template thematic analysis Theoretical model: ground-up, qualitative descriptive approach Methodology: not stated	Interviews via telephone at place of convivence for participant Focus groups held in metropolitan Perth	No. 15 patients’ community conversation No. 13 patient interview No. 13 focus group (3 carer) Gender: stated women in community conversation Ethnicity: not reported Age: not reported Treatments: not reported	Six key themes emerged regarding various aspects of illness and treatment experiences described by the women and their careers. Health-Related Quality of Life (HRQOL) was contextual themes. Post diagnosis and treatment-related issues, relationships and supports with family and friends, financial issues, relationships with healthcare providers, and self-perceived coping strategies were the key themes identified
Chi et al. (2024) USA Pennsylvania—Pittsburgh	Investigate factors that influence information seeking behaviours and information avoidance behaviours and information resources among women with Ovarian Cancer and their caregivers	Method: individual telehealth interviews Analysis: inductive and deductive coding methodologies Theoretical model: not stated Methodology: not stated	Conducted remotely due to COVID-19 between May and November 2022. Semi-structured lasting 60 min	No. 20 (18 women and 2 family caregiver) Gender: 1 male, 19 female Age: 100% 18 years or over Ethnicity: 100% white Treatments: 50% had finished treatment and in remission	The study provided perceptions of 18 women with ovarian cancer and 2 family caregivers on their health information seeking behaviours, focusing on their self- reported factors of active information seeking, passive information acquisition, and information avoidance. Furthermore, this is the first study to identify their preferred information sources of active information seeking and passive information acquisition
Dumas et al. (2021) UK London	Explore the lived experience of older patients with advanced ovarian cancer undergoing chemotherapy, their treatment preferences, and treatment burden	Methods: focus groups and interviews Analysis: inductive thematic analysis Theoretical model: phenomenological qualitative study Methodology: qualitative	Semi structure interview schedule. Focus groups 2 h Interview 29–60 min	No. 15 patients Gender: 100% female Age: 65 or older Ethnicity: did not discuss Treatments: had completed at least three cycles of chemotherapy	The older women in this study were overwhelmingly positive about their experience of cancer care and desire for anticancer treatment, despite facing treatment burden and therapy-related toxicities. Older women may face additional challenges in terms of information retention and managing medical comorbidities. Additional methods of delivering information could be useful to improve patient centred decision making
Galica et al. (2020) Canada Cancer centre of Southern Ontario in Kingston, Ontario, Canada	To explored how ovarian cancer survivors living in small urban and rural areas cope with FCR	Methods: semi-structure interviews Analysis: descriptive qualitative design using thematic analysis Theoretical model: qualitative descriptive study Methodology: qualitative	Focus groups or 1:1 telephone interviews and the Fear of Cancer Recurrence Inventory (FCRI) to collect data	No. 15 patients Gender: 100% female Age: 100% over 18 Ethnicity: 93% white, 7% mixed Treatments: was not allowed to have radiation to the brain	Fear of cancer recurrence was a concern for most ovarian cancer survivors who used a variety of ways to cope. Results can be used to guide nurses’ discussions with post-treatment ovarian cancer survivors or be used to inform refinement and development of resources to assist ovarian cancer survivors living in small urban and rural settings to cope with FCR
Han et al. (2021) USA Portland, ME	To explore the relationship between prognostic uncertainty and FCR among patients with ovarian cancer	Methods: individual in-depth interviews a convenience sample Analysis: inductive qualitative analysis Theoretical model: not stated Methodology: qualitative	Semi-structured interviews lasting 40–60 min	No. 21 patients Gender: 100% female Age: 100% over 18 Ethnicity: 100% white Treatments: had completed first line treatment with surgery and/or chemotherapy	Nearly all of whom reported experiencing significant FCR, which they traced to an awareness of the possibility of a bad outcome. Some participants valued and pursued prognostic information as a means of coping with this awareness, suggesting that prognostic uncertainty causes FCR. However, most participants acknowledged fundamental limits to both the certainty and value of prognostic information and engaged in various strategies aimed not at reducing but constructing and maintaining prognostic uncertainty as a means of sustaining hope in the possibility of a good outcome
Harris et al. (2024) UK Manchester, UK	Explore advanced ovarian cancer patients’ experience of surgery and identify areas in which quality of life may be impacted	Methods: semi-structure interview Analysis: inductive approach to thematic analysis outlined via the 6 steps in Braun and Clarke Theoretical model: not stated Methodology: qualitative	Semi-structured telephone or face to face interviews lasting 33–68 min	No. 20 patients Gender: 100% female Age: 100% over 18 Ethnicity: 17 British 1 White and Black African 1 Chinese 1 Other White background Treatments: all had surgery	Five key themes emerged: (1) care services; (2) experiences of a stoma; (3) preoperative experience; (4) impact of surgery; and (5) coping mechanisms. This research explored the ways in which a patient’s quality of life is impacted by surgery and highlights areas in which further support may be needed. Knowledge of the patient experience may also aid decision-making for both clinicians and patients when considering different treatment pathways
Jelicic et al. (2018) AUS NSW, VIC, QLD, SA, WA ACT, NT	To explore the healthcare experiences and preferences of women with ovarian cancer during this phase, and identify opportunities to enhance women’s experiences and outcomes	Methods: semi structure interviews Analysis: qualitative descriptive approach Theoretical model: inductive thematic analysis Methodology: essentialist qualitative approach	Semi-structure interview lasting 60 min, online and via telephone	No. 34 patients Gender: 100% female Age: 100% over 18 years Ethnicity: not disclosed Treatments: 6 months post-diagnosis	Five key themes, with 10 subthemes. Themes included (1) navigating uncertainty, (2) responsiveness in healthcare, (3) relational communication, (4) patient‐centred information, and (5) preparation for living beyond cancer treatment. Providing appropriate and accessible information to assist women to prepare for life beyond cancer treatment may be facilitated through the holistic consideration of patients’ biopsychosocial health, both in the short and long term
Chou et al. (2019) Taiwan MacKay Memorial Hospital in Taipei,	To explore the lived experiences of Taiwanese patients diagnosed with ovarian cancer who have received at least one cycle of IP chemotherapy	Methods: semi-structured interview Analysis: qualitative content analysis Theoretical model: descriptive qualitative Methodology: qualitative	1:1 interview	No. 9 patients Gender: 100% female Age: 100% 20 years or older Ethnicity: not stated Treatments: had received at least one cycle of IP chemotherapy	The side effects of IP chemotherapy may be more challenging than those experienced with IV chemotherapy; IP chemotherapy administration requires advanced knowledge of treatment regimens and symptom management interventions
Lee et al. (2000) Malaysia Gynaecologic Day Unit at the University of Malay Medical Centre (UMMC)	This study aimed to explore the coping strategies employed by women with recurrent ovarian cancer in Malaysia, a developing multicultural country in Asia	Methods: interviews—questions based on topic guide Analysis: thematically Theoretical model: not stated Methodology: qualitative	Interview lasting 30–45 min	No.10 patients Gender: 100% female Age: 100% over age 18 Ethnicity: Malay, Chinese, and Indian Treatments: with recurrence of ovarian cancer and on chemotherapy	Six coping strategies were identified: (1) maintaining a mindset of hopefulness, (2) avoidance of information, (3) accepting their condition, (4) seeking spiritual help, (5) relying on family for support, and (6) coping with financial costs. Coping strategies employed during ovarian cancer recurrence in this setting were rarely based on the accurate information appraisal, but rather on the individual emotion and personal beliefs
Lian et al. (2022) USA UAB, Alabama, Birmingham	Obtain perspectives from ovarian cancer patients on job demands, cancer demands, and workplace or cancer resources and strategies to manage the cancer-work interface using the cancer-work management conceptual framework	Methods: interviews Analysis: inductive thematic analysis Theoretical model: not discussed Methodology: qualitative	Interviews conducted via telephone and in-person	No. 22 patients Gender: 100% female Age: 100% over 18 Ethnicity: 36% Black 68% Treatments: ovarian cancer patient receiving systemic therapy who screened positive for financial distress	Cancer care teams should consider screening patients for employment concerns; streamline care to minimise the side effects, time, and transportation demands of treatment on patients and caregivers; maximise utilisation of available resources; and proactively communicate with employers to accommodate patients and caregivers who want or need to work
Matsui et al. (2024) Japan Hirakata, Osaka, Japan	This study was aimed to assess the nuanced transformation of sexuality in Japanese women after ovarian cancer treatment	Methods: interview, questions based on a theoretical framework Analysis: continuous comparative analysis Theoretical model: threefold realities—Eriksons Methodology: modified grounded theory approach	Two face-to-face or online interviews were conducted for each participant	No. 18 patients Gender: 100% female Age: 100% over the age of 20 Ethnicity: Japanese Treatments: Hx of ovarian cancer and have undergone initial treatment for ovarian cancer. Those who are part of a patient association	The analysis revealed five categories and 13 subcategories that encapsulated the transformation of sexuality in women with ovarian cancer. These categories included (1) confronting the reality of losing their ovaries and uterus, (2) contemplating the reversibility and irreversibility of womanhood, (3) grappling with altered and often negative feelings toward sexual activity, (4) reassessing the essence of partnership, and (5) finding contentment in their identity as women. The transformation of sexuality in Japanese women undergoing treatment for ovarian cancer unfolds in five distinct stages. This evolution appears to be influenced by the unique characteristics of ovarian cancer diagnosis and treatment, past reproductive decisions, communication dynamics with partners, and societal norms in Japan
Moskalewicz et al. (2022) Poland Poznan	To explore lived time of women with ovarian cancer during chemotherapy	Methods: semi-structured and consisted of a set question Analysis: consensual qualitative research—not Giorgi phenomenology, inductive coding Theoretical model: qualitative phenomenological exploration Methodology: qualitative	Interview conducted at home, hotel, and hospital lasting 45–90 min	No. 9 patients Gender: 100% female Age: 100% over 18 Ethnicity: not commented on Treatments: ovarian cancer and having chemotherapy over 6 months. One excluded due to being in love	The ten temporal themes identified across the interviews are (1) regret and guilt, (2) distant happy past, (3) living in the now, (4) explicit passing of time, (5) chemo-clock, (6) paradox of time, (7) short temporal horizon, (8) losing and gaining control over time, (9) unpredictability, and (10) finitude and death
Newell et al. (2025) USA Washington, DC	The lived experiences of OC patients with sleep disturbance and fatigue and the range of factors that they perceive as contributing to these symptoms	Methods: individual semi-structured interview Analysis: iterative deductive-inductive approach, axial coding Theoretical model: not described Methodology: qualitative	Interviews in person after 64 participants had filled in a survey around sleep disturbance	No. 20 patients Gender: 100% Female Age: 100% over 18 years Ethnicity: 70% white 10% Black or African American 15% Latino or Hispanic 5% Multiple race Treatments: III and IV OC diagnosed and treated within 3 years	Findings underscore that while sleep disturbance and fatigue are intense among participants with OC, the lived experiences of these symptoms are qualitatively distinct at different points during treatment. Participants’ dissatisfaction with providers’ communication suggests the need for improved screening and scaled interventions for advanced OC patients
Pozzar et al. (2019) USA Massachusetts and Southern New Hampshire, USA	To describe the cancer care process as it is perceived by women with ovarian cancer	Methods: data were collected via individual interviews with participants Analysis: thematic analysis Theoretical model: grounded theory Methodology: qualitative	How it occurred: interviews, questions designed open ended. Range 40–90 min	No. 18 patients Gender: 100% female Age: 100% over 18 Ethnicity: 17 White, 1 Asian Treatments: have a diagnosis of ovarian cancer	The findings from this study suggest that although women with ovarian cancer are motivated to preserve their physical health, psychosocial factors, such as communication, support, and self-concept, may also affect decision making. To ensure that patient-centred care is a priority in the ovarian cancer care setting, these findings can inform future efforts to promote guideline-concordant treatment and the adoption of novel treatment therapies
Roche et al. (2016) USA John Hopkins Hospital, Maryland, USA	Explored women’s experiences with navigating the healthcare system during treatment for ovarian cancer	Methods: focus groups and one on one interviews Analysis: inductive thematic analysis and coded ATLAS,ti software Theoretical model: not discussed Methodology: qualitative	Focus groups of three or more. Moderated by trained facilitators	No. 16 patients Gender: 100% female Age: 100% over 18 2 public 9 private Ethnicity: 13 White 3 African American Treatments: ovarian cancer and had > 9 months from treatment initiation	Systems-based challenges were perceived as burdens to ovarian cancer survivors at our institution. The role of a consistent, accessible care team and efficient delivery of resources in the care of women with ovarian cancer should be explored further
Smith et al. (2024) USA Pennsylvania	To examine patient barriers and facilitators to PARP inhibitor (PARP-I) maintenance therapy in ovarian cancer	Methods: interviews Analysis: exploratory descriptive analysis Theoretical model: grounded theory approach Methodology: qualitative	Open ended interview questions, participants were paid for their time	No. 10 patients Gender: 100% female Age: 100% over 18 Ethnicity: 8 White 1 Black 1 Asian Treatments: eligible if they spoke English or Spanish, had been prescribed PARP-I for OC	Patients paid on average $227.50 monthly for PARP-I, straining resources for some participants. While sampled patients were insured, all patients identified having no or inadequate insurance as a major barrier to PARP-I. At the same time, all participants prioritised clinical effectiveness over costs of care. Patients identified PARP-I delivery from specialty pharmacies, separate and different from other medications, as a potential barrier, but each had been able to navigate delivery. Patients expressed significant initial side effects of PARP-I as a potential barrier yet reported clinician communication and prompt dose reduction as facilitating continuation
Staneva et al. (2019) AUS Brisbane, Queensland, AUS	To explore women’s accounts of the factors they believed were helpful during their ovarian cancer treatment	Methods: interviews Analysis: Thematic analysis Theoretical model: critical realist framework Methodology: qualitative	In-depth semi-structured interviews via telephone lasting between 35 and 90 min	No. 18 patients Gender: 100% female Age: 100% over 18 years Ethnicity: 14 White 2 Asian 2 Other Treatments: chemotherapy for ovarian cancer within 2 years	Three main themes related to women’s experiences of dealing with chemotherapy: “optimistic tenacity”, which illustrates a specific stoic identity that women assumed during treatment; “self-care”, which reflects the health behaviours and activities women engaged in and lifestyle adjustments they made; and “support systems”, which emphasises the importance of social, emotional, and medical support and the specific needs shared by women undergoing treatment for ovarian cancer. Our findings contribute to a deeper understanding of women’s unique experiences of treatment that may influence whether they complete chemotherapy for ovarian cancer
Stilos et al. (2018) Canada Sunnybrook Health Sciences Centre	To explore the experience of family caregivers caring for their loved ones with advanced ovarian cancer	Methods: semi-structured interviews Analysis: not reported Theoretical model: not reported Methodology: qualitative descriptive	Interviews ranged from 45 to 90 min in length. A semi-structured interview guide was developed	No. 13 caregivers Gender: 12 female 1 male Age: 100% over 18 years Ethnicity: English speaking Treatments: care to have occurred within 3 years since diagnosis	Family caregivers play a vital role in caring for loved ones with advanced ovarian cancer. It is crucial that the HCT understands the caregiver experience to optimally support patients and their family caregivers. In this study, family caregivers identified several gaps in care including insufficient information, lack of communication at transition points, lack of guidance in navigating the healthcare system, and limited access to palliative supports. To help bridge these gaps in care, guidelines exist to outline the management of ovarian cancer and to support all members of the interprofessional oncology team along the disease trajectory
Tan et al. (2020) AUS All across AUS Urban 70.8% Rural 28.9% Unknown 0.5%	To synthesise the experiences of individuals and their caregivers through a thematic analysis of their responses	Methods: online survey conducted in 2017 Analysis: thematic analysis—using inductive, micro analytic approach Theoretical model: not described Methodology: qualitative	Survey sent out by Ovarian Cancer Australia	No. 288 ovarian cancer 78 caregivers Gender: 68 female 10 male Age: 100% 18 years and over Ethnicity: all fluent in English Treatments: diagnosed with ovarian cancer or being a caregiver	For both groups, the uncertainty created at diagnosis led to a cascade of complex responses. For the individuals, uncertainty gave rise to fears for the future, which were exacerbated by unmet healthcare needs or treatment‐related difficulties. For some individuals, these fears led to disruption to their lives, isolation, and emotional distress. For others, helpful coping styles and social support protected them from these negative consequences. For caregivers, the processes were similar, but uncertainty predominantly led to feelings of hopelessness and “survivor guilt”
Tetteh et al. (2017) USA North west Ohio, USA	To explore the effects of ovarian cancer and treatments on women’s sexual self-concept and how factors such as age, stage of cancer, and level of social support intersect to shape the meaning women construct of their experiences	Methods: Interviews and focus groups Analysis: Iterative Theoretical model: Grounded theory Methodology: Qualitative	Semi-structured interviews, 45–120 min, took place face to face and via telephone plus focus groups	No. 28 patients Gender: 100% female Age: 100% over 18 Ethnicity: 27 white 1 Mexican American Treatments: Any stage of OC journey	The results showed that ovarian cancer and its treatments affect women’s understanding of their sexual self-concept. Their understanding is also influenced by life conditions at the time of diagnosis, the treatment regimen, and factors such as age and level of social support. Thus, sexual self-concept in the context of ovarian cancer needs to be reconceptualised to account for how the disease presents itself
Thomas et al. (2018) USA Located on twitter	To understand the supportive care needs of women with ovarian care at the end of treatment	Methods: Twitter chat Analysis: Simpler signals quantitative data analysis – descriptive content analysis for qualitative data Theoretical model: Digital content analysis Methodology: Qualitative and Quantitative	1 h timeframe over twitter chat (300 unique tweets)	No. 103 during twitter chat Gender: Not stated Age: Not stated Ethnicity: Not stated Treatments: Anyone with Ovarian Cancer. Advertised to the public social media community (#gyncsm)	The chat occurred over a 1-h time frame on Twitter and resulted in more than 300 unique and original tweets from 43 participants during the chat and an additional 60 unique participants following the chat. Survivors and physicians represented 32% and 11% of participants, respectively; caregivers, advocates, and other clinicians represented the remaining participants. Participants noted deep interest in receiving support during survivorship and dissatisfaction with currently available resources. Sentiment analysis showed that participants viewed the support from social media in a positive light and also revealed negative sentiment around the lack of support from healthcare providers at the end of treatment
Tsai et al. (2020) Taiwan Taipei, Taiwan	The lived experiences of ovarian cancer survivors amid the disease trajectory and psychosocial adaptation	Methods: semi-structured interview Analysis: not discussed Theoretical model: Giorgi’s phenomenological Methodology: qualitative	Face to face interview	No. 21 patients Gender: 100% female Age: 100% over 20 years Ethnicity: not stated Treatments: ovarian cancer survivor, age 20 and over, mandarin or Taiwan, without psychiatric disorder	Three themes and 12 subthemes were extracted: (1) a depressed state, as if facing a fierce enemy, (2) shadow of cancer recurrence, (3) a change of mindset to move forward. The conventional models caring for patients with ovarian cancer are based on disease and unable to meet their needs because the lengthy rehabilitation journey. Therefore, medical personnel should emphasise patients’ medical autonomy and combine professional care and social resources to help patients developing adjustment strategies and establishing support systems in timely manner for body, mind, and soul of these patients
Webb et al. (2023) AUS Sydney, AUS—recruited from all over AUS	To explore experiences of FCR among caregivers of people with ovarian cancer	Methods: semi-structure interview Analysis: thematic analysis Theoretical model: not discussed Methodology: qualitative and quantitative	Recruited through Ovarian Cancer Australia—survey completed then interview via telephone	No. 24 caregivers Gender: 54% male 46% female Age: 100% over 18 Ethnicity: not discussed Treatments: providing care for partner, friend or family member with ovarian cancer	Caregivers supporting people with ovarian cancer experience worries and concerns related to cancer recurrence or progression. These experiences are conceptually different to survivor experiences. Fear of one’s family member dying, and the dual nature of caregiver protection/self‐protection mean it is imperative that interventions are tailored specifically to caregiver needs. Future research facilitating the development of appropriate measures and interventions is essential to reduce caregiver FCR
Williams et al. (2025) AUS	To explore clinical trials awareness, information access and participation among Australians with ovarian cancer	Methods: online focus groups and interviews Analysis: inductive content analysis—conventional content analysis Theoretical model: exploratory qualitative approach Methodology: qualitative	Online focus group or one on one interview, lasting 67 min and interview 34 min State or city: all NSW	No. 21 patients Gender: 100% female Age: 100% over 18 Ethnicity: 100% Australian Treatments: diagnosis of ovarian, fallopian tube, or peritoneal cancer	Five themes and five subthemes emerged. In theme 1, “barriers exist that affect clinical trial awareness and participation”. In theme 2, “instigating the conversation and doing my own research” was necessary to access clinical trials. Theme 3 “finding solutions to improve clinical trial awareness and information access”. Theme 4 explained that “altruism is a motivator” in willingness to participate in trials. In theme 5, “emotions regarding clinical trials are varied”. These qualitative insights will inform development of a cross-sectional survey for national distribution among Australians with ovarian cancer. Results will assist in developing solutions to improve clinical trials awareness and information access
Yan et al. (2022) USA Texas Austin, USA	The genetic testing–related information needs of patients with OC to inform the design of interactive technology-based interventions that can enhance communication of genetic testing information to patients	Methods: interview Analysis: inductive and deductive approach to qualitative content analysis Theoretical model: not discussed Methodology: qualitative	Demographic questionnaire, semi-structured interview, and a co-design session. Interview lasting 40–2 h. Researcher held debrief after each interview to generate main themes	No. 20 patients Gender: 100% female Age: 100% over 18 Ethnicity: 16 White 1 American Indian 1 Mexican American 2 not reported Treatments: diagnosed with ovarian cancer and undergone genetic testing	Patients with OC need a range of information to address the uncertainties and challenges that they encounter while taking genetic tests. Their preferences for channels to receive information vary widely. A multichannel information delivery solution that combines both provider-led and peer-to-peer education models is needed to supplement existing genetic counselling to effectively meet the genetic testing–related information needs of patients with OC

**Table 2:**
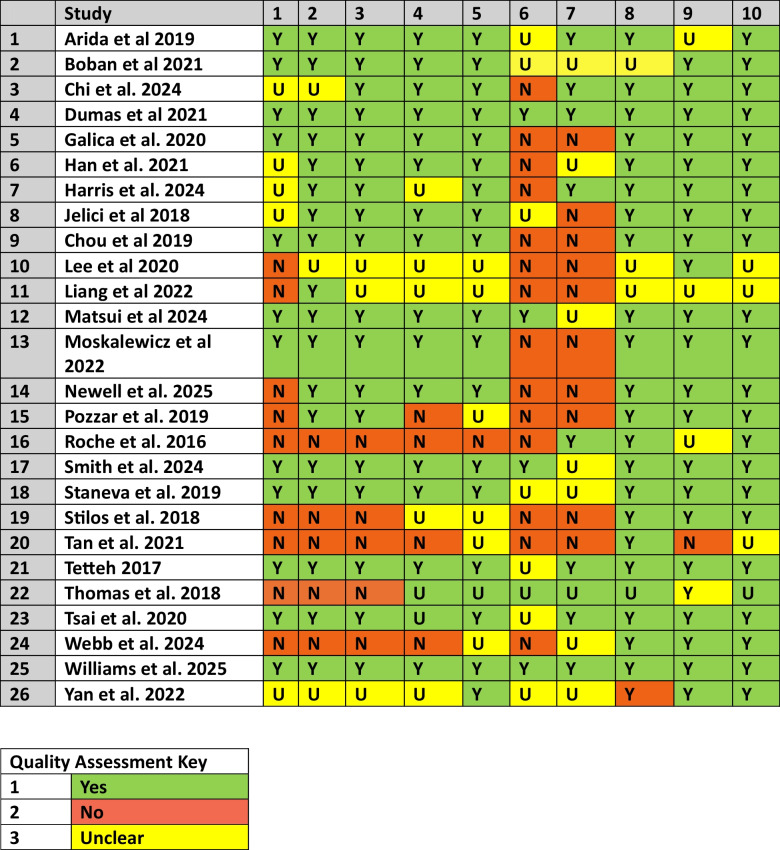
Results of Quality Assessment Study

Item number check list key*: (1) Is there congruity between the stated philosophical perspective and the research methodology? (2) Is there congruity between the research methodology and the research question or objectives? (3) Is there congruity between the research methodology and the methods used to collect data? (4) Is there congruity between the research methodology and the representation and analysis of data? (5) Is there congruity between the research methodology and the interpretation of results? (6) Is there a statement locating the researcher culturally or theoretically? (7) Is the influence of the researcher on the research, and vice-versa, addressed? (8) Are participants, and their voices, adequately represented? (9) Is the research ethical according to current criteria for recent studies, and is there evidence of ethical approval by an appropriate body? (10) Do the conclusions drawn in the research report flow from the analysis, or interpretation, of the data?

### Findings

A total of 133 individual findings (114 unequivocal, 19 credible) were identified, and there were 5 unsupported, as shown in Supplementary file 4. Of these, four main synthesised findings were entitled: (1) awareness around ovarian cancer, (2) communication in the healthcare sector, (3) everything that comes with the disease, and (4) what the future holds; see Table 3.

**Table 3:** Systematic review synthesised findings (134 findings)

Findings synthesised finding	Categories	Synthesised finding
1PT, 4PT, 47PT, 48PT, 49PT, 52PT, 71PT, 87PT, 105PT, 119PT, 131PT	• Knowledge about ovarian cancer • Symptoms awareness • Advocacy	**Awareness around ovarian cancer** Patients spoke about the symptoms being vague and a lack of awareness around the warning sign in relation to ovarian cancer. There were quotes of diagnostic delay due to lack of awareness from health professional and several participants spoke about intentionally ignoring their symptoms due to work and family commitments. Although not many, some patients spoke about their GP being aware and ordering appropriate screening test and referring onto a specialist in a timely manner. Many patients spoke about the advocacy and work that needs to continue to bring awareness to ovarian cancer
7PT, 9PT, 12PT, 13PT, 14PT, 15PT, 16PT, 17PT, 18PT, 19PT, 20PT, 21Pt, 22PT, 23PT, 24PT, 26PT, 50PT, 84PT, 88PT, 90PT, 107PT, 118PT, 120PT, 127PT, 128PT, 129PT,	• Communication between healthcare professional and the patient • Understanding the information • Who is my team?	**Communication in the healthcare sector** Many participants felt that there were communication gaps between the healthcare system, particularly during treatment. Some participants shared perspectives that their healthcare providers did not provide clear, transparent information that was easy to understand, leading them to seek information on their own. Numerous participants spoke about a need for clear guidelines for follow-up treatment and confusion by being told contradictory information between health specialists. Many participants wanted clear handouts for who their treating team members were as it was confusing as the specialist all did different things There were quotes of satisfied communication that arose from clear explanation, handouts easy to understand and being a part of the process by being asked for their opinion. Generally patient although frequently feeling overwhelmed by the volume of information felt they had excellent care and support and expressed sincere gratitude. “It’s like a blanket around you isn’t it”
2PT, 3PT, 5PT, 6PT, 8PT, 25PT, 27PT, 28PT, 31PT, 33PT, 40PT, 41PT, 42PT, 43PT, 44PT, 45PT, 51PT, 53PT, 54PT, 55PT, 56PT, 59PT, 60PT, 62PT, 63PT, 65PT, 66PT, 67PT, 68PT, 70PT, 75PT, 76PT, 81PT, 82PT, 83PT, 91PT, 92PT, 93PT, 94PT, 95PT, 96PT, 97PT, 98PT, 102CG, 103CG, 104CG, 108PT, 109PT, 113CG, 114PT, 115PT, 116PT, 117PT, 121PT, 123CG, 124CG, 125CG, 126CG,	• Caregivers being part of the journey as well • Side effects from treatment • Sexuality health supportive care needs	**Everything that comes with the disease** Caregivers’ voices spoke about a feeling of helplessness and wanting to be involved in care but not knowing where to get information from. Many of the caregivers went on websites to reach out for further information. Being asked how they are going while their family member was going through the cancer journey was not common. Caregivers tried to keep a positive mindset and have minimal long-term plans as they felt like life was in limbo. Many caregivers held their feelings in as not to put worry on their loved ones as there was already significant stress in life The patients also had many hardships while going through the cancer journey. Financial and fertility stress were spoken about with fatigue being the biggest hurdle. Sexuality and intimacy were spoken about as hardships with a disconnect between the GP and specialised treating team as to who provides this care. Chemotherapy side effects were spoken about in length around hair loss and pain. This area of care seemed to be managed well in the postoperative and post chemotherapy period. Although menopause education remained a debate as to some participants having supportive care post and many still missing key information around this topic
10PT, 11PT, 29PT, 30PT, 32PT, 34PT, 35PT, 36PT, 37PT, 38PT, 39PT, 46PT, 57PT, 58PT, 61PT, 64PT, 69PT, 72PT, 73PT, 74PT, 77PT, 78PT, 79PT, 80PT, 85PT, 86PT, 99PT, 100PT, 101CG, 110PT, 111PT, 122PT, 130PT, 132PT, 133PT, 134PT	• Support through lived experience • Community and family support	**What the future holds** Patients voiced their need for social support through lived experience groups. They spoke about how When somebody tells you their story, you can pick up their strength and maybe improve your own strengths. It’s a tough fight”. Family and friends gave patients the courage to fight fit. Nearly all patients and caregivers spoke about the need to keep moving forward by being strong and to keep going. The fear of recurrence was always there, and nearly all participants spoke about it, with family and friends being key confidence in this area to speak to

### Synthesised finding 1: awareness about ovarian cancer

There was a total of 10 findings across these studies [2, 6, 17–23] that supported this synthesised finding.

### Knowledge about ovarian cancer

Many of the studies [2, 6, 17, 18, 22, 23] reported experiences about presenting symptoms of ovarian cancer being vague and a lack of knowledge around ovarian cancer. There was limited knowledge around the warning signs. When diagnosis was made, many patients were emotionally shocked as they had put the symptoms down to other things [6, 17, 18].

I came to my own conclusion about what stage 4 meant. You know nobody really explained what we (might) need? What to expect? What’s coming next? I feel like we got none of that information at all (Caregiver, pg.466) [23]

I thought my menopause had begun, but my period came back, and it would not stop. I thought it was a common gynaecologic [issue], similar to excessive vaginal discharge. (Patient, Pg. 102) [18]

I really didn’t have any symptoms before I was diagnosed either. I’d just moved to Virginia like 6 to 8 months before. I had a newborn. I stopped nursing 2 weeks before I was diagnosed. I had a kid that just started kindergarten. I was just getting used to it; I was tired, but I had 3 little kids 5 and under. And, um, I noticed that my belly was getting a little bit bigger. I didn’t have any discharge. I didn’t have back pain. I didn’t have any bloating. I didn’t have any of that stuff. And um, one day, I just did a little twist thing, and I could feel somethings swish. (Patient, Pg. 58) [17]

### Symptom awareness

Several patients delayed seeing a GP by ignoring their presenting symptoms. Some experienced delays in diagnosis due to work or family commitments taking priority or having minimal symptoms that prompted them not to be seen immediately by their GP [1, 17]. Many participants conveyed their difficulties in finding a GP who felt their symptoms warranted further investigation [17]. Women often reported that they felt that their GPs often brushed off their symptoms or attributed their signs and symptoms to a less serious alternative diagnosis [17]. Inevitably, this resulted in feelings of frustration and disappointment about an avoidable delay in their diagnosis and, therefore, limited their curative treatment options available to them. When concerns were validated by their GPs in a timely manner, the majority of participants articulated that referrals transitioned promptly without delays [17].

When I first noticed my symptoms, I guess I was just a little bit bewildered, but I thought very little of it, I actually thought I may have been developing irritable bowel syndrome, because I felt really well only I had to run to the bathroom more often than I ever have before … I just simply thought something might be a little bit funny and I, but I really thought it was not serious. (Patient, Pg. 1) [17]

Oh I just said I had felt bloated, and my stomach wasn’t feeling right, and she the new [GP] just gave me an external examination and could feel it and said right I need you to go and have scans done and x-rays done and things like that. So I did that the next day and had to go back and see her that was on a Tuesday and a Thursday and by that time she had made an appointment for me to see a gynaecologist on the Friday. So everything moved very quickly. (Patient, Pg. 3) [17]

### Advocacy

Most participants, both caregivers and patients, spoke about the importance of self-advocacy and how their needs were often not advocated for. There is a growing need for further research and awareness around ovarian cancer. One participant compared ovarian cancer to breast cancer and expressed their frustration in relation to what they saw as the inequitable financial burden between both tumours [2, 21].

Ovarian cancer definitely needs money, and I always get a little bit titchy when breast cancer gets so much. I keep saying, what about me? What about ovarian cancer?” (Patient, Pg. 5) [2]

It was my lack of knowledge of the signs and symptoms of ovarian cancer … ensure as many women as possible realize what these signs and symptoms are and that they need to be very proactive. (Patient, Pg. 215) [21]

If I was able to read how advanced ovarian cancer fared or what their experiences were I think that would have prepared me for what I thought she could have been going through to relate, and I might have been able to find that somewhere on the Net, but nobody was offering it, that information directly to us (Caregiver, pg.466) [23]

### Synthesised finding 2: communication in the healthcare sector

Overall, many participants encountered communication challenges either with their GP or with navigating their care providers across both GP and their cancer specialist. A total of 29 findings [1, 2, 6, 20–22, 24–28] supported this synthesised finding.

### Communication between healthcare professionals and patients

Most participants reported feeling there were fundamental communication gaps in the healthcare system that let them down, particularly during treatment. A lack of written information and difficulties in understanding verbal communication resulted in some participants “googling” key words after medical appointments to seek clarification of medical jargon [1, 24].

Because of my complex medical problem, I’ve been out for a few months affected by surgery and by several treatments. So, I found that (hospital’s) communication between the different departments just wasn’t there. (Patient, Pg. 38) [1]

When I got the original test results on MyChart, there was a bunch of words that I wasn’t really sure what it meant. So, I looked up the words and it said that it was an ovarian tumour. (Patient, Pg. 3) [24]

Alternatively, some participants and caregivers had positive relationships with their healthcare provider, describing how being involved and explaining “everything” made them in control. Inviting patients to share their thoughts and emotions fostered more meaningful and effective communication with their healthcare provider [24].

He’ll [doctor] sit down and explain everything, and if I don’t understand it, he’ll go back over it, and they may give me a paper to tell me what the side effects of the chemo is and everything. And the doctor asks for my opinion, what I think, and he is wonderful. (Patient. Pg. 4) [24]

### Understanding the information

Information overload was described many times and the complexity to understand what was relevant and what was not became emotionally overwhelming for some participants. On the other hand, some participants wanted to collect as much information as possible and be involved in every step of the decision-making process. Most participants continued to express concerns about communication breakdowns and the complexity of identifying their cancer providers and establishing contact. Many wanted change in having clear handouts with dedicated contact numbers that were answered by the specialist team when needed [6, 20, 24].

Just layman terms are great with me. If it gets too complicated, then I get frustrated, and then I get more confused, and then I have to look up more words. And by the time I’m done, I’m getting totally upset. (Patient. Pg. 5) [24]

What I found helpful was when I went in with the results of the CT scan there was an oncologist sitting in there and also a nurse telling me of the physical implications of the surgery, I was about to have … Well, that was really helpful, and frightening at the same time. (Patient, Pg. 6) [6]

They should give you an idea on paper who is your exact team, who you contact for different things. [Having a care team] also allows when you do go and see the doctor, to focus on the things that need to be focused on. (Patients, Pg. 977) [20]

I found communications were very confusing at first. Who to talk to, you know, to call anytime. You know, ‘this is the number’, and then you might phone that number, you might make it a callback, and then you might get someone you don’t recognize. (Patients, pg.977) [20]

### Who is my team?

The patients felt it important that their team was with them as a central point of contact, and they found it distressing to feel like another number in the healthcare system. Consensus was that half had support lacking, and the other half expressed gratitude for their specialist team [20, 25, 27]. Generally, participants felt they had excellent care and support from the cancer centre and expressed sincere gratitude.

It’s like a blanket around you isn’t it. (Patient, Pg. 7) [25]

They should give you an idea on paper who is your exact team, who you contact for different things. [Having a care team] also allows when you do go and see the doctor, to focus on the things that need to be focused on. (Patients, Pg. 977) [20]

It began to feel … like an assembly line. Like, come in, number 1,024. Mark her off. Reminding me that I’m gonna die, just in case I didn’t catch it. I said, ‘If I go to another hospital, they may not be as well renowned, but maybe they will listen to me and maybe they’ll fight for me’. Because [at this hospital] … I feel like no one is fighting for me. They’re just waiting for me to die. (Patients, Pg. 598) [27]

### Synthesised finding 3: everything that comes with the disease

Caregivers and patients both spoke of the feeling of helplessness post-diagnosis. Side effects and hardships were a challenge faced not only by patients but also by their loved ones. Caregivers discussed how they wanted to be involved but did not know “how”. A total of 57 findings [1, 2, 6, 7, 17–19, 22, 23, 25, 26, 29–36] provided insight and evidence to gain an understanding of what it is like to live with ovarian cancer.

### Caregivers being part of the journey as well

Informal caregivers spoke about minimal support and a feeling of hopelessness from the clinical team and would have valued timely access to more emotional care or have this care provided to them during clinical consultations. Of the 58 findings informing this synthesised finding, there were nine from informal caregivers [23, 33].

When my mom was getting sicker, I was kind of waiting for them to say, ‘I how are you managing at home?’ I And I felt I often had to take the ball in my own hands, isn’t there any seat we can put on the toilet, a shower chair, a bedrail to help my mom? (Caregiver, Pg. 466) [23]

My concerns for myself are nothing compared to what your partner’s going through. You know, so you sort of think I’ll just keep a lid on it or keep it all nice and calm and stuff… because of that attitude you don’t see any build‐up of emotions and things like that. (Caregiver, Pg. 8) [33]

### Side effects from treatment

Surgical and chemotherapy side effects were spoken about in length. Women frequently highlighted the profound impact of hair loss and physical pain as key concerns in everyday life. The management of these side effects was vaguely addressed by their care treating team during the immediate postoperative period; however, over time, the support and supported self-management fell away, and they were left to grapple with the side effects completely on their own. One patient spoke about the physical stoma being more traumatic than their abdominal wound, and there was a complete lack of support to cope psychologically with the changes in body image and self-management strategies from their care team [1, 7, 32].

It was the stoma that was mentally and physically like more traumatic than the actual abdominal wound and surgery itself. (Patient, Pg. 4) [7]

I had more energy when I was going through chemo… and even after the surgery, I was up and walking 2 days after the surgery… But it’s the [VEGF inhibitor] and the [PARP inhibitor] that have created the fatigue that have created the weakness. The biggest side effect would be fatigue. I’ve never had fatigue like that before. (Patients, Pg. 45) [32]

I guess it was not even initially when I wasn’t told about certain things I could access like my super. I had to find out I think two years down the track or something. So it wasn’t, nobody even gave me that sort of information. (Patient, Pg. 38) [1]

### Sexual health supportive care needs

Sexuality and fertility stress were spoken about with fatigue being the biggest hurdle and impacts on quality of life and adjustment. Sexuality and intimacy were spoken about as hardships with a disconnect between the GP and cancer specialist treating team as to who navigates and provides this rehabilitation care. Many patients spoke about postoperative health information being suboptimal, with a focus on surgically induced menopause as a key gap in discharge information support. Infertility resulted in profound feelings of loss among the participants, and one patient stated that it was the first time they felt this level of psychological burden in their life [18, 31].

[I would have preferred] just a bit more information on menopause, surgical menopause … because it didn’t even cross my mind, I’m quite aware that when your ovaries are gone, you know that is your menopause well and truly, but at the time I needed to be refreshed about that … it was something that didn’t even enter my mind until afterwards. And I actually asked the gynaecologist, I said to them ‘are you sure you didn’t leave a swab in there?’ I said ‘because I’ve got these sweats really bad’ and he just said ‘I’ll put you on a tablet’ and it was good, it all just disappeared. But if I had have known ahead I could have asked for that straight off. (Patient, Pg. 7) [18]

Not being able to carry a child … I felt a sense of loss for the first time at that point. (Patient, Pg. 5) [31]

My partner is still in his 40 s, but he can no longer engage in sexual activity, and I feel sorry for him and a great sense of responsibility. I think that I should do something, but even that has become tiring. (Patient, Pg. 6) [31].

### Synthesised finding 4: what the future holds

A total of 37 findings [7, 18, 19, 21, 23, 24, 26–31, 36–38] supported the synthesised finding of people with ovarian cancer’s future outlook on life.

### Support through lived experience

Patients voiced their need for social support through lived experience groups. They spoke about how listening and being around others with lived experience can improve their own strength and resilience. “It’s a tough fight” [29]. Ovarian cancer peer support groups were used as a coping strategy to move through difficult times [7, 18, 27, 29, 30].

[My friends] never really reached out to me … so I reached out to [the hospital]. But they didn’t really have a support group for post-chemo patients. I just decided, ‘You know what, I’m gonna just deal with this myself’. I weeded out a lot of people. (Patients, Pg. 598) [27]

… it helps you, you know, talking to people who are going through cancer. (Patient, Pg. 5) [7]

### Community and family support

Family and friends gave patients the strength, courage, and unconditional emotional support to cope with cancer, treatments, and associated side-effects. Nearly all patients and caregivers spoke about the need to keep moving forward, reflecting on the past, but to remain “future focussed”. Many people with ovarian cancer articulated the impact of fear of cancer recurrence, and this stressor also impacted family and friends who were identified as the key people who provided that needed support. Many patients also spoke about the importance of God in providing them with strength in coping during the challenges of cancer, and the spiritual guidance provided to them from being Christian. They found strength knowing that their faith and grace from God were supporting them during life’s most difficult moments in their current season of their life, when they had no one else to turn to [18, 29, 30].

One participant (Patient 5) viewed even the smallest possibility of a best-case outcome as a reason for hope: ‘even if there was a 1% chance, I was like it didn’t matter.’ (Patient, Pg. 4) [29]

Like I said, I have to think positive. So that when the thing (ovarian cancer) happens to us again, we won’t feel so down, won’t feel it so much. At the most, 1 or 2 days only, you know. (Patient, Pg. 44) [18]

The social worker told me that love is courage. When I used positive thoughts to replace rage, anger, and guilt, [I felt like] my immune system would be activated. That was a huge encouragement, and I began to encourage myself. (Patient, Pg. 103) [18]

I’m a Christian, so my life is in God’s hands …I’m in good hands. So I’m at peace, I don’t worry … so God gives that strength. If not, emotionally and spiritually, I really want to give up. (Patient, Pg. 44) [30]

## Discussion

To our knowledge, this is the first systematic review to provide an understanding of the supportive care needs among people affected by ovarian cancer and their informal caregivers within the current contemporary clinical landscape. The findings show how supportive care needs of women and their informal caregivers are still not being met in all holistic aspects of health and wellbeing, despite regular follow-up with cancer healthcare professionals.

The included 26 studies that investigated supportive care needs varied widely in research design, setting, population, and targeted outcomes. This variation enabled maximum understanding from a wide variety of diverse viewpoints and clinical settings to clearly identify existing shortcomings in care provision. There were several publications [39] which examined the entire continuum of supportive care, whereas other studies examined treatment-specific impacts, for example, during chemotherapy [19] and access to and awareness of clinical trials among women [2].

Almost all studies included in this systematic review confirmed women’s lack of awareness and education about the signs and symptoms of ovarian cancer. The findings resonate with the research conducted by Reid [4] who identified that women have on average four or more symptoms prior to diagnosis and do not receive timely or appropriate investigations. This important finding underscores the need for public health strategies and education for healthcare professionals in the community setting to ensure ovarian cancer is managed in a safe and timely manner. Our findings also point out that many of the women had very limited or no knowledge that ovarian cancer existed, and this observation is in keeping with Reid’s [4]. Evidence has shown that over two-thirds of women had no prior knowledge of the disease before diagnosis [4]. However, when women had a higher level of prior knowledge surrounding the disease, they were more likely to visit the GP within 3 months of presenting symptoms [4].

Women reported that they needed to change their GP clinician to another GP provider to ensure that their worrying and distressing symptoms were investigated, as they were being dismissed or clinically mismanaged [6]. Commonly, GPs would misdiagnose ovarian cancer signs and symptoms for other conditions, such as menopausal symptoms or bloating symptoms for dietary intolerance, or they would commence Hormone Replacement Therapy [6]. The evidence has been documented many times that early detection at an earlier stage of ovarian cancer results in longer life expectancy and improved health outcomes for people affected by this disease [4].

Communication and understanding of medical terminology are imperative language tools that can hinder or help women and their informal caregivers in navigating this health trajectory. The findings emphasised the need for improvement in this critical area. Women frequently described the difficulty that they experienced in understanding medical “jargon” and how this exchange led to confusion. Many women would have valued clear self-management handouts and easy-to-understand information in relation to their local community support networks. It was important that women knew who to contact when they had further questions that they required to be answered by cancer healthcare professionals. Suboptimal clinician-patient information exchange is not new and aligns to previously published research [40]. Consequently, it means that this aspect of supportive care should be a future focus of health service provision improvement [41]. Furthermore, Sharkiya [42] reiterates how verbal and non-verbal communication strategies have a direct positive impact on patient-centred outcomes.

This review has highlighted the enormity of the informal caregiver’s role as the support person for those affected by cancer. Albeit there was little evidence available to fully understand the breadth of impact that cancer had on the informal caregiver. Our findings illustrate a notable lack in informal caregiver support, and many informal caregivers did not have emotional support available to them throughout the cancer care continuum. As it pertains to informal caregivers, it is recommended that future research should explore their unique experiences to guide future support provision [43]. Molassiotis [43] describes in a recent scoping review that when people diagnosed with cancer have informal caregiver support available to them, the person with cancer has better coping capabilities and healthier lifestyle habits. While the person with cancer has better coping capabilities and healthier lifestyle habits, the informal caregiver remains poorly if at all supported by healthcare professionals.

Side effects of cancer treatment, both physical and psychological, were highlighted frequently by women and their informal caregivers as major hurdles to manage. Often, they were provided with limited supported self-management to cope and recover because of cancer and its treatments [44]. Moreover, our data highlight how physical support like stoma care and postoperative pain relief were prioritised more than emotional support.

Community connection was important for both women and informal caregivers as they felt an emotional benefit from connecting with others going through the same health trajectory. By connecting with like-minded people who have experienced similar life paths, stress was reduced and a greater outlook on life was achieved [44]. Peer support was relevant for both women and informal caregivers; therefore, an emphasis should be put on the importance of accessing support groups at the beginning of an ovarian cancer diagnosis.

## Limitations

This review followed a transparent and rigorous process as per the protocol registered with PROSPERO and followed the Joanna Briggs Institute (JBI) meta-aggregation methodology [12]. However, only studies published in the English language were included; therefore, by omission, the findings of this review may not be transferrable to the non-English speaking community. This systematic review notes that out of the 133 findings, only nine were from the informal caregivers, identifying a critical gap in the literature.

## Conclusion

This systematic review has provided a new insight into the supportive care needs of women living with ovarian cancer and their informal caregivers. Importantly, this evidence synthesis has provided a valuable foundation upon which future improvement in service design and redesign can be driven forward in partnership with healthcare providers and cancer consumers.

Research efforts should focus on driving forward (1) improved awareness around ovarian cancer signs and symptoms, (2) tailored supported self-management, (3) increased information and support for informal caregivers throughout the cancer care continuum, and (4) embedding cancer recovery with a focus on wellness.

## Supplementary Information

Below is the link to the electronic supplementary material.

ESM1 (DOCX 29.9 KB)

ESM2 (DOCX 31.2 KB)

ESM3 (DOCX 45.5 KB)

ESM4 (DOCX 169 KB)

## Data Availability

No datasets were generated or analysed during the current study.

## References

[1] Boban S et al (2021) Women diagnosed with ovarian cancer: patient and carer experiences and perspectives. Patient Relat Outcome Meas 12:33–43 33623464 10.2147/PROM.S272688 PMC7896761

[2] Williams N, Russell H, Bradhurst B (2025) Exploring clinical trials awareness, information access and participation amongst Australians with ovarian cancer: a qualitative study. Support Care Cancer 33(3):176 39934363 10.1007/s00520-025-09221-2 PMC11814028

[3] Dilley J et al (2020) Ovarian cancer symptoms, routes to diagnosis and survival - population cohort study in the ‘no screen’ arm of the UK Collaborative Trial of Ovarian Cancer Screening (UKCTOCS)*.* Gynecol Oncol 158(2):316–322 32561125 10.1016/j.ygyno.2020.05.002 PMC7453382

[4] Reid F et al (2021) The world ovarian cancer coalition every woman study: identifying challenges and opportunities to improve survival and quality of life. Obstet Gynecol Surv 76(5):275–276 10.1136/ijgc-2019-000983 32540894

[5] O’Hanlan KA, Gordon JC, Sullivan MW (2018) Biological origins of sexual orientation and gender identity: impact on health. Gynecol Oncol 149(1):33–42 29605047 10.1016/j.ygyno.2017.11.014

[6] Jelicic L et al (2019) Experiences and health care preferences of women with ovarian cancer during the diagnosis phase. Psychooncology 28(2):379–385 30485590 10.1002/pon.4952

[7] Harris E et al (2024) Advanced ovarian cancer patients’ experiences of surgical treatment: a qualitative analysis. Semin Oncol Nurs 40(4):151679 38890076 10.1016/j.soncn.2024.151679

[8] Paterson C et al (2023) What are the unmet supportive care needs of people affected by cancer: an umbrella systematic review. Semin Oncol Nurs 39(3):151353 36435657 10.1016/j.soncn.2022.151353

[9] Herbert S-L et al (2025) Supportive care and information needs in relation to quality of life among patients with breast cancer and gynaecological cancer during the time of treatment. Arch Gynecol Obstet 311(2):467–479 39576340 10.1007/s00404-024-07805-7 PMC11890328

[10] van der Kruk SR et al (2022) Psychosocial well-being and supportive care needs of cancer patients and survivors living in rural or regional areas: a systematic review from 2010 to 2021. Support Care Cancer 30(2):1021–1064 34392413 10.1007/s00520-021-06440-1 PMC8364415

[11] Hodges LJ, Humphris GM, Macfarlane G (2005) A meta-analytic investigation of the relationship between the psychological distress of cancer patients and their carers. Soc Sci Med 60(1):1–12 15482862 10.1016/j.socscimed.2004.04.018

[12] Lockwood C, Munn Z, Porritt K (2015) Qualitative research synthesis: methodological guidance for systematic reviewers utilizing meta-aggregation. JBI Evid Implement 13(3):179–187 10.1097/XEB.0000000000000062 26262565

[13] Wormald R, Evans J (2018) What makes systematic reviews systematic and why are they the highest level of evidence? Ophthalmic Epidemiol 25(1):27–30 28891724 10.1080/09286586.2017.1337913

[14] Page MJ et al (2021) The PRISMA 2020 statement: an updated guideline for reporting systematic reviews. BMJ 372:n71 33782057 10.1136/bmj.n71 PMC8005924

[15] Capoluongo E et al (2017) Guidance statement on BRCA1/2 tumor testing in ovarian cancer patients. Semin Oncol 44(3):187–197 29248130 10.1053/j.seminoncol.2017.08.004

[16] Purssell E, Gould D (2021) Undertaking qualitative reviews in nursing and education - a method of thematic analysis for students and clinicians. Int J Nurs Stud Adv 3:100036 38746709 10.1016/j.ijnsa.2021.100036 PMC11080565

[17] Arida JA et al (2019) Mothering with advanced ovarian cancer: “you’ve got to find that little thing that’s going to make you strong”. Cancer Nurs 42(4):E54–E60 29489476 10.1097/NCC.0000000000000550 PMC6111003

[18] Chou JF, Lu Y (2019) Intraperitoneal chemotherapy: the lived experiences of Taiwanese patients with ovarian cancer. Clin J Oncol Nurs 23(6):E100-e106 31730595 10.1188/19.CJON.E100-E106

[19] Moskalewicz M, Popova Y, Wiertlewska-Bielarz J (2022) Lived time in ovarian cancer - a qualitative phenomenological exploration. Eur J Oncol Nurs 56:102083 34998214 10.1016/j.ejon.2021.102083

[20] Long Roche K et al (2016) Little big things”: a qualitative study of ovarian cancer survivors and their experiences with the health care system. J Oncol Pract 12(12):e974–e980 27601509 10.1200/JOP.2015.007492 PMC6366291

[21] Tan JH, Sharpe L, Russell H (2021) The impact of ovarian cancer on individuals and their caregivers: a qualitative analysis. Psychooncology 30(2):212–220 32940943 10.1002/pon.5551

[22] Thomas TH et al (2018) The needs of women treated for ovarian cancer: results from a #gyncsm Twitter chat. J Patient-Cent Res Rev 5(2):149–157 31413999 10.17294/2330-0698.1592 PMC6664330

[23] Stilos K et al (2018) Exploration of families’ experiences caring for loved ones with advanced ovarian cancer. J Hosp Palliat Nurs 20(5):464–470 30188440 10.1097/NJH.0000000000000463

[24] Chi Y et al (2024) Women with ovarian cancer’s information seeking and avoidance behaviors: an interview study. JAMIA Open. 10.1093/jamiaopen/ooae011 38384330 10.1093/jamiaopen/ooae011 PMC10881099

[25] Dumas L et al (2021) Exploring older women’s attitudes to and experience of treatment for advanced ovarian cancer: a qualitative phenomenological study. Cancers 13(6):1207 33801991 10.3390/cancers13061207 PMC8001330

[26] Galica J et al (2020) Coping with fear of cancer recurrence among ovarian cancer survivors living in small urban and rural settings: a qualitative descriptive study. Eur J Oncol Nurs 44:101705 32006720 10.1016/j.ejon.2019.101705

[27] Pozzar RA, Berry DL (2019) Preserving oneself in the face of uncertainty: a grounded theory study of women with ovarian cancer. Oncol Nurs Forum 46(5):595–603 31424458 10.1188/19.ONF.595-603

[28] Tsai LY, Tsai JM, Tsay SL (2020) Life experiences and disease trajectories in women coexisting with ovarian cancer. Taiwan J Obstet Gynecol 59(1):115–119 32039777 10.1016/j.tjog.2019.11.032

[29] Han PKJ et al (2021) Cause or effect? The role of prognostic uncertainty in the fear of cancer recurrence. Front Psychol 11:626038 33519656 10.3389/fpsyg.2020.626038 PMC7843433

[30] Lee YK et al (2021) Coping strategies among Malaysian women with recurrent ovarian cancer: a qualitative study. Asia Pac J Oncol Nurs 8(1):40–45 33426188 10.4103/apjon.apjon_38_20 PMC7785070

[31] Matsui R, Aoki S, Seto N (2024) A qualitative analysis of sexual transformation in Japanese women after ovarian cancer treatment. Asia-Pac J Oncol Nurs 11(4):100381 38495644 10.1016/j.apjon.2024.100381 PMC10944108

[32] Newell A et al (2025) Patients with advanced ovarian cancer and their experiences and perceptions of sleep disturbance and fatigue across the treatment trajectory: a qualitative study. Gynecol Oncol 198:42–48 40411968 10.1016/j.ygyno.2025.05.011

[33] Webb K et al (2024) Fear of cancer recurrence in ovarian cancer caregivers: A qualitative study. Psychooncology 33(1):e6255 38047732 10.1002/pon.6255

[34] Tetteh DA (2017) I feel different”: ovarian cancer and sexual self-concept. women’s reproductive health 4(1):61–73

[35] Smith AJB et al (2024) Having cancer is very expensive”: a qualitative study of patients with ovarian cancer and PARP inhibitor treatment. Gynecol Oncol 186:170–175 38691987 10.1016/j.ygyno.2024.04.018

[36] Liang MI et al (2022) Navigating job and cancer demands during treatment: a qualitative study of ovarian cancer patients. Gynecol Oncol 166(3):481–486 35902296 10.1016/j.ygyno.2022.07.021 PMC10910482

[37] Staneva AA et al (2019) I wasn’t gonna let it stop me”: exploring women’s experiences of getting through chemotherapy for ovarian cancer. Cancer Nurs. 10.1097/NCC.0000000000000574 29538021 10.1097/NCC.0000000000000574

[38] Zhang Y et al (2022) Understanding the information needs of patients with ovarian cancer regarding genetic testing to inform intervention design: interview study. JMIR Cancer 8(1):e31263 35133282 10.2196/31263 PMC8864522

[39] Primeau C et al (2024) Patient experiences of patient–clinician communication among cancer multidisciplinary healthcare professionals during “breaking bad news”: a qualitative systematic review. Semin Oncol Nurs 40(4):151680 38918149 10.1016/j.soncn.2024.151680

[40] Evans Webb M et al (2021) The supportive care needs of cancer patients: a systematic review. J Cancer Educ 36(5):899–908 33492650 10.1007/s13187-020-01941-9 PMC8523012

[41] Feo R, Kitson A, Paterson C (2025) How the caring life-course theory can enhance the central role of communication across the cancer care continuum. Semin Oncol Nurs. 10.1016/j.soncn.2025.152019 40992969 10.1016/j.soncn.2025.152019

[42] Sharkiya SH (2023) Quality communication can improve patient-centred health outcomes among older patients: a rapid review. BMC Health Serv Res 23(1):886 37608376 10.1186/s12913-023-09869-8 PMC10464255

[43] Molassiotis A, Wang M (2022) Understanding and supporting informal cancer caregivers. Curr Treat Options Oncol 23(4):494–513 35286571 10.1007/s11864-022-00955-3 PMC8918600

[44] Schulman-Green D et al (2008) Quality of life among women after surgery for ovarian cancer. Palliat Support Care 6(3):239–247 18662417 10.1017/S1478951508000497 PMC3648854

