# Supplementary material for: The experience and supportive care needs in people affected by ovarian cancer and their informal caregivers: a qualitative systematic review
Source: Support Care Cancer. 2026 Mar 23;34(4):354. doi: 10.1007/s00520-026-10542-z (PMC13009085; doi:10.1007/s00520-026-10542-z)
Supplement: Supplementary file 2 — (DOCX 31.2 KB) [file 520_2026_10542_MOESM2_ESM.docx]

**Supplement 2 – Database searches**

The CINAHL (via EBSCOhost), Google Scholar (via Publish or Perish 8), Medline (via EBSCOhost), PsycINFO (via EBSCOhost), Scopus, and Web of Science Core Collection databases, were searched on 5 August 2025 to locate relevant studies. Search terms, limiters, and number of search results by database:

**CINAHL via EBSCOhost**

| Search | Concept | Search Terms | Results |
| --- | --- | --- | --- |
| #1 | “Sample” – People affected by Ovarian cancer. | (ovar* N2 (cancer* OR neoplasm* OR tumo#r*)) OR (MH "Ovarian Neoplasms+") | 22,139 |
| #2 | “Phenomenon of Interest” – experiences, needs, and preferences for supportive care. | care OR need* OR support* OR unmet* OR (MH "Needs Assessment") | 2,662,019 |
| #3 | “Research Type” – qualitative studies. | (((("semi-structured" OR semistructured OR unstructured OR informal OR "in-depth" OR indepth OR "face-to-face" OR structured OR guide) N3 (interview* OR discussion* OR questionnaire*)) OR “appreciative inquiry” OR “case stud*” OR ethnograph* OR fieldwork OR "field work" OR “focus group*” OR “grounded theory” OR observation* OR phenomenology OR qualitative) OR (MH "Qualitative Studies+")) | 672,551 |
| #4 | Experiences and supportive care needs in people affected by ovarian cancer. | #1 AND #2 AND #3 | 645 |
|  |  | Limiter: English language studies.  Limiter: 2015- | 367 |

**Google Scholar via Publish or Perish 8**

| Search | Concept | Search Terms | Results |
| --- | --- | --- | --- |
| #1 | Experiences and supportive care needs in people affected by ovarian cancer. | ovarian-cancer \| experiences \| care \| qualitative  Limiter: 2015- | 45 |

**MEDLINE via EBSCOhost**

| Search | Concept | Search Terms | Results |
| --- | --- | --- | --- |
| #1 | “Sample” – People affected by Ovarian cancer. | (ovar* N2 (cancer* OR neoplasm* OR tumo#r*)) OR (MH "Ovarian Neoplasms+") | 138,418 |
| #2 | “Phenomenon of Interest” – experiences, needs, and preferences for supportive care. | care OR need* OR support* OR unmet* OR (MH "Needs Assessment+") | 7,557,873 |
| #3 | “Research Type” – qualitative studies. | (((("semi-structured" OR semistructured OR unstructured OR informal OR "in-depth" OR indepth OR "face-to-face" OR structured OR guide) N3 (interview* OR discussion* OR questionnaire*)) OR “appreciative inquiry” OR “case stud*” OR ethnograph* OR fieldwork OR "field work" OR “focus group*” OR “grounded theory” OR observation* OR phenomenology OR qualitative) OR (MH "Qualitative Studies+")) | 1,892,151 |
| #4 | Experiences and supportive care needs in people affected by ovarian cancer. | #1 AND #2 AND #3 | 1,827 |
|  |  | Limiter: English language studies.  Limiter: 2015- | 1,092 |

**PsycINFO via EBSCOhost**

| Search | Concept | Search Terms | Results |
| --- | --- | --- | --- |
| #1 | “Sample” – People affected by Ovarian cancer. | (ovar* N2 (cancer* OR neoplasm* OR tumo#r*)) | 1,475 |
| #2 | “Phenomenon of Interest” – experiences, needs, and preferences for supportive care. | care OR need* OR support* OR unmet* | 1,896,065 |
| #3 | “Research Type” – qualitative studies. | (((("semi-structured" OR semistructured OR unstructured OR informal OR "in-depth" OR indepth OR "face-to-face" OR structured OR guide) N3 (interview* OR discussion* OR questionnaire*)) OR “appreciative inquiry” OR “case stud*” OR ethnograph* OR fieldwork OR "field work" OR “focus group*” OR “grounded theory” OR observation* OR phenomenology OR qualitative)) | 754,176 |
| #4 | Experiences and supportive care needs in people affected by ovarian cancer. | #1 AND #2 AND #3 | 239 |
|  |  | Limiter: English language studies.  Limiter: 2015- | 116 |

**Scopus**

| Search | Concept | Search Terms | Results |
| --- | --- | --- | --- |
| #1 | “Sample” – People affected by Ovarian cancer. | (ovar* W/2 (cancer* OR neoplasm* OR tumo#r*)) | 168,284 |
| #2 | “Phenomenon of Interest” – experiences, needs, and preferences for supportive care. | care OR need* OR support* OR unmet* | 17,015,471 |
| #3 | “Research Type” – qualitative studies. | (((("semi-structured" OR semistructured OR unstructured OR informal OR "in-depth" OR indepth OR "face-to-face" OR structured OR guide) W/3 (interview* OR discussion* OR questionnaire*)) OR “appreciative inquiry” OR “case stud*” OR ethnograph* OR fieldwork OR "field work" OR “focus group*” OR “grounded theory” OR observation* OR phenomenology OR qualitative)) | 6,043,979 |
| #4 | Experiences and supportive care needs in people affected by ovarian cancer. | #1 AND #2 AND #3 | 2,873 |
|  |  | Limiter: English language studies.  Limiter: Document type - Article  Limiter: 2015- | 1,492 |

**Web of Science Core Collection**

| Search | Concept | Search Terms | Results |
| --- | --- | --- | --- |
| #1 | “Sample” – People affected by Ovarian cancer. | (ovar* NEAR/2 (cancer* OR neoplasm* OR tumo$r*)) | 141,146 |
| #2 | “Phenomenon of Interest” – experiences, needs, and preferences for supportive care. | care OR need* OR support* OR unmet* | 10,075,395 |
| #3 | “Research Type” – qualitative studies. | (((("semi-structured" OR semistructured OR unstructured OR informal OR "in-depth" OR indepth OR "face-to-face" OR structured OR guide) NEAR/3 (interview* OR discussion* OR questionnaire*)) OR “appreciative inquiry” OR “case stud*” OR ethnograph* OR fieldwork OR "field work" OR “focus group*” OR “grounded theory” OR observation* OR phenomenology OR qualitative)) | 4,168,451 |
| #4 | Experiences and supportive care needs in people affected by ovarian cancer. | #1 AND #2 AND #3 | 1,739 |
|  |  | Limiter: English language studies.  Limiter: Document type – Article/Early access  Limiter: 2015- | 860 |
