# Supplementary material for: The experience and supportive care needs in people affected by ovarian cancer and their informal caregivers: a qualitative systematic review
Source: Support Care Cancer. 2026 Mar 23;34(4):354. doi: 10.1007/s00520-026-10542-z (PMC13009085; doi:10.1007/s00520-026-10542-z)
Supplement: Supplementary file 3 — (DOCX 45.5 KB) [file 520_2026_10542_MOESM3_ESM.docx]

| Author | Title | Reason |
| --- | --- | --- |
| Frey et al. 2014 | A qualitative study of ovarian cancer survivors' perceptions of endpoints and goals of care | Out of date |
| Alimujiang et al. 2019 | "I am not a statistic" ovarian cancer survivors' views of factors that influenced their long-term survival | Wrong outcomes |
| Alinejad-Mofrad et al. 2021 | Spousal sexual life issues after gynaecological cancer: a qualitative study | Wrong patient population |
| Ammon et al. 2024 | Deciphering the needs of patients with hereditary breast and ovarian Cancer in the Process of Genetic Counselling to Inform the Development of a Mobile Support App: a qualitative study in Germany | Wrong patient population |
| Asiedu et al. 2018 | "Ultimately, mom has the call": Viewing clinical trial decision making among patients with ovarian cancer through the lens of relational autonomy | wrong focus |
| Butow et al 2014 | Caring for women with ovarian cancer in the last year of life: a longitudinal study of caregiver quality of life, distress and unmet needs | Out of date |
| Bal et al. 2013 | Sexual health in patients with gynaecological cancer: A qualitative study | out of date |
| Boding et al. 2025 | '… but I live in hope …' how the term 'survivor' impacts identity and feelings of inclusivity in survivorship services following ovarian cancer treatment | wrong focus |
| Breistig et al. 2019 | Healthcare personnel as a source of comfort in recurrent ovarian cancer | not in english |
| Burles et al. 2013 | "Cautiously optimistic that today will be another day with my disease under control": understanding women's lived experiences of ovarian cancer | out of date |
| Burles et al. 2013 | “Cautiously Optimistic That Today Will Be Another Day With My Disease Under Control” | duplicate |
| Butlin et al. 2023 | Searching for wisdom: A phenomenological investigation of women's perspectives following participation in an ovarian cancer supportive care group | dissertation |
| Chi et al. 2021 | Challenges of Ovarian Cancer Patient and Caregiver Online Health Information Seeking | duplicate |
| Crook et al. 2015 | Connecting patients, researchers and clinical genetics services: the experiences of participants in the Australian Ovarian Cancer Study (AOCS) | wrong focus |
| DellaRipa et al. 2015 | Perceptions of Distress in Women with Ovarian Cancer | out of date |
| DellaRipa et al. 2014 | Distress in Women with Ovarian Cancer | dissertation |
| De-Rooij et al. 2018 | Survivorship care planning in gynaecologic oncology-perspectives from patients, caregivers, and health care providers | Wrong patient population |
| Ekwall et al. 2011 | Patients' perceptions of communication with the health care team during chemotherapy for the first recurrence of ovarian cancer | Out of date |
| Ekwall et al. 2014 | Lived experiences of women with recurring ovarian cancer | out of date |
| Elit et al. 2010 | It's a choice to move forward: women's perceptions about treatment decision making in recurrent ovarian cancer | out of date |
| Frey et al. 2014 | A qualitative study of ovarian cancer survivors' perceptions of endpoints and goals of care | out of date |
| Gleeson et al. 2013 | Communication and Information Needs of Women Diagnosed With Ovarian Cancer Regarding Treatment-Focused Genetic Testing | out of date |
| G-Pinar et al. 2015 | Problematic Areas Related to Sexual Life of Individuals with Gynaecological Cancer: A qualitative Study in Turkey | Wrong patient population |
| Guenther et al. 2014 | A Phenomenological Approach to Describe the Lived Experience of Ovarian Cancer | out of date |
| Guenther et al. 2012 | The lived experience of ovarian cancer: A phenomenological approach | out of date |
| Güler et al. 2021 | Feelings, thoughts and experiences of women diagnosed with adnexal mass: A qualitative study | Wrong patient population |
| Hagan et al. 2013 | Ovarian Cancer Survivors' Experiences of Self-Advocacy: A Focus Group Study | out of date |
| Hagan et al. 2016 | Patient Education vs. Patient Experiences of Self-advocacy: Changing the Discourse to Support Cancer Survivors | wrong focus |
| Holland-Hart et al. 2025 | Participants' perspectives of the advanced ovarian cancer biomarker study VALTIVE1: a qualitative study | wrong focus |
| Holliday et al. 2018 | Experience and Expectations of Ovarian Cancer Patients in Australia | Wrong study design |
| Jayde et al. 2013 | The experience of chemotherapy-induced alopecia for Australian women with ovarian cancer | out of date |
| KB Roland et al. 2013 | A literature review of the social and psychological needs of ovarian cancer survivors | review |
| Kennedy et al. 2022 | 'We do need to keep some human touch'—Patient and clinician experiences of ovarian cancer follow‐up and the potential for an electronic patient‐reported outcome pathway: A qualitative interview study | Wrong patient population |
| Ketcher et al. 2021 | Attributions of survival and methods of coping of long-term ovarian cancer survivors: a qualitative study | wrong focus |
| Kyriacou et al. 2017 | Fear of cancer recurrence: A study of the experience of survivors of ovarian cancer | Wrong patient population |
| Lopez et al. 2019 | Age-related supportive care needs of women with gynaecological cancer: A qualitative exploration | Wrong patient population |
| Maheu et al. 2015 | Breast and ovarian cancer survivors' experience of participating in a cognitive-existential group intervention addressing fear of cancer recurrence | Wrong patient population |
| Morrell et al. 2012 | The perils of a vanishing cohort: a study of social comparisons by women with advanced ovarian cancer | out of date |
| Moskalewicz et al. 2021 | "From chemo to chemo"-the temporal paradox of chemotherapy | wrong focus |
| Mujumdar et al. 2021 | A qualitative study on the impact of long-distance travel for gynaecologic cancer care | Wrong patient population |
| NicGiollaChomhaill et al 2024 | Fear of recurrence in women with ovarian cancer: A qualitative evidence synthesis | review |
| Noll et al. 2022 | Building opportunities to improve quality of life for women with ovarian cancer in low- and middle-income countries: the Every Woman Study | wrong focus |
| Oshima et al. 2011 | A Qualitative Study of Japanese Patients' Perspectives on Post-treatment Care for Gynaecological Cancer | out of date |
| Polen-De et al. 2021 | Advanced ovarian cancer patients identify opportunities for prehabilitation: A qualitative study | wrong focus |
| Poort et al. 2021 | Lived experiences of women reporting fatigue during PARP inhibitor maintenance treatment for advanced ovarian cancer: A qualitative study | wrong focus |
| Pozzar et al. 2018 | Patient, physician, and caregiver perspectives on ovarian cancer treatment decision making: lessons from a qualitative pilot study | Wrong patient population |
| Puppo et al. 2019 | Cancer survivors providing care: A call for a new approach | Wrong study design |
| Ridgeway et al. 2017 | Patient and family member perspectives on searching for cancer clinical trials: A qualitative interview study | Wrong patient population |
| Saggu et al. 2025 | 'I was eager to do anything I could to improve the situation': a qualitative study of patients' experiences and views of prehabilitation for ovarian cancer surgery | wrong focus |
| Schulman-Green et al. 2012 | One step at a time: self-management and transitions among women with ovarian cancer | out of date |
| Scott Finlayson et al. 2017 | The Experience of Being Aware of Disease Status in Women with Recurrent Ovarian Cancer: A Phenomenological Study | dissertation |
| Seeratan et al. 2024 | Patient-reported outcome measures (PROMs) to personalise follow-up care of ovarian cancer: what do patients think? A qualitative interview study | wrong focus |
| Seibaek et al. 2012 | Hoping for the best, preparing for the worst: the lived experiences of women undergoing ovarian cancer surgery | out of date |
| Tsai et al. 2020 | Lived experiences in the illness trajectory for elderly patients with ovarian cancer in Taiwan: A phenomenological study | full text not available |
| Vogel et al. 2018 | A qualitative study of barriers to genetic counseling and potential for mobile technology education among women with ovarian cancer | wrong focus |
| Wainer et al. 2012 | The treatment experiences of Australian women with gynaecological cancers and how they can be improved: A qualitative study | out of date |
| Walker et al. 2010 | Supportive expressive group therapy for women with advanced ovarian cancer | out of date |
| Williams et al. 2013 | Capturing the patient's experience: using qualitative methods to develop a measure of patient-reported symptom burden: an example from ovarian cancer | out of date |
| Wilmoth et al 2011 | Ovarian Cancer Survivors: Qualitative Analysis of the Symptom of Sexuality | out of date |
| Yaman et al. 2016 | Psychological Problems Experienced by Women with Gynaecological Cancer and How They Cope with It: A Phenomenological Study in Turkey | Wrong patient population |

Table 3: Excluded Full text with reason
