# Supplementary material for: The experience and supportive care needs in people affected by ovarian cancer and their informal caregivers: a qualitative systematic review
Source: Support Care Cancer. 2026 Mar 23;34(4):354. doi: 10.1007/s00520-026-10542-z (PMC13009085; doi:10.1007/s00520-026-10542-z)
Supplement: Supplementary file 4 — (DOCX 169 KB) [file 520_2026_10542_MOESM4_ESM.docx]

**Supplementary Table 4. Study findings and illustrations**

| **Study: Name & Year** |  | | **Evidence** | | | **Label** |
| --- | --- | --- | --- | --- | --- | --- |
|  | **Finding** | **Qualitative Illustrations** | **Unequivocal** | **Credible** | **Not supported** |  |
| **Arida et al. 2019** | Being diagnosed | Women described their memories of being on the precipice of a major life-changing event in which their former, relatively healthy self becomes a self that is ill with cancer as they described the symptoms and experiences leading up to seeking medical care and receiving a diagnosis of ovarian cancer:  One day, my defining moment (which probably all of you had) was standing looking in the mirror at Kohl’s; I was shopping with my daughter, and she’s in the next dressing room, and I said I’m not buying any more clothes; this is ridiculous I look like I’m pregnant all the time, and I pushed on my stomach, and I thought this doesn’t feel like fat anymore, and it was just looking in that mirror at that one moment you know okay, now I’ve got to get to the bottom of this; something is wrong. (Patient, Pg 58)  I really didn’t have any symptoms before I was diagnosed either. I’d just moved to Virginia like 6 to 8 months before. I had a newborn. I stopped nursing 2weeks before I was diagnosed. I had a kid that just started kindergarten. I was just getting used to it; I was tired, but I had 3 little kids 5 and under. And, um, I noticed that my belly was getting a little bit bigger. I didn’t have any discharge. I didn’t have back pain. I didn’t have any bloating. I didn’t have any of that stuff. And um, one day, I just did a little twist thing, and I could feel somethings swish. (Patient, Pg 58) | **X** |  |  | 1.Patient |
|  | Becoming a patient | Women spoke of the challenges presented by a multitude of symptoms and adverse effects related to their disease and treatment:  Nausea a can get to you. They have awesome drugs, awesome drugs that help with that. But that was annoying. I never got sick-sick; I just didn’t feel good sometimes. And the kids were at home, so I was motivated to get off the couch and not lay around. (Patient, Pg 58)  I mean I can’t even get up and get my kids dressed, I’m so tired. (Patient, Pg 58) |  | **x** |  | 2.Patient |
|  | Mothering with cancer | As women embraced the identity of striving to balance the competing demands of motherhood and being a cancer patient, they spoke of numerous modifications and adaptations, such as relying on extended family and friends in new ways:  Them watching me go through it. Uhhh, awful. Watching my 80-year-old parents take me to chemo and having to deal with their baby being sick. And just knowing what she has and how bad it can be. I think that is hard. Very hard. I think it is hard to watch. Kids seeing their mummy. It’s tough. (Patient, Pg 58)  Yes, but you know what? It’s really cool. I’ve got really incredible neighbours okay? And they had formed my own team. (Patient, Pg 58) | **X** |  |  | 3.Patient |
| **Boban et al. 2021** | Diagnosis, Treatment and Related Issues | Four factors were identified in relation to the symptomatic presentation pertaining to the disease. Participants experienced pre-diagnostic symptoms including abdominal/bowel discomfort and pain, urinary urgency, fatigue, weight gain, abnormal menstrual bleeding and/or menopausal symptoms. Lack of awareness of disease symptoms by both patients and health professionals (HPs) was a related issue. Due to work and family commitments, several participants intentionally ignored their symptoms. In further, majority of the participants expressed diagnostic delay as another challenge faced during their clinical diagnosis phase. |  |  | **X** | 4. Patient |
|  | Relationships and Support | All participants agreed that relationships with their family and friends influenced their lives. Some participants spoke of experiencing lack of support with unpredictable reactions and withdrawal of family and/or friends. Other participants spoke of being avoided and noticed that people around them “react differently” which then created emotional reactions such as upset and insecurity. Furthermore, sexual relations and a changed level of intimacy with a partner/spouse were identified as an important subtheme in their lives. |  |  | **X** | 5. Patient |
|  | Financial Issues | Some mentioned the financial toxicity associated with their illness and that they lacked knowledge of how to access support services such as paying the bills without going into debt and having to access their superannuation funds for urgent and necessary expenses:  “I guess it was not even initially when I wasn’t told about certain things I could access like my super. I had to find out I think two years down the track or something. So it wasn’t, nobody even gave me that sort of information.” (Patient, Pg 38) |  | **X** |  | 6. Patient |
|  | Interaction with Health Professionals | Overall, many participants felt there were communication gaps in the health care system, particularly during treatment, and participants experienced various forms of communication challenges either with or between oncologists and GPs and specialist departments:  “Because of my complex medical problem, I’ve been out for a few months affected by surgery and by several treatments. So, I found that (hospital’s) communication between the different departments just wasn’t there.” (Patient, Pg 38) |  | **X** |  | 7. Patient |
|  | Coping Strategies | Participants expressed that by being independent and knowing their innermost selves provided them motivation and strength throughout their lives. In addition, providing self-encouragement through positive attitude and feeling gratitude helped them.  “I do need and want to practice gratitude every day. I am grateful for what I’ve got. And I’m much more in tune with the little things in life.” (Patient, Pg 38) |  | **X** |  | 8. Patient |
| **Chi et al. 2024** | **Active information seeking:**  Filling knowledge gaps | Participants indicated that they would consciously proceed to seek information when they identified a gap in their knowledge. For example, P16 said: “my platelets were too low to get chemo, and I looked up and saw what kind of foods you could eat to help bring it up.” (Patient. Pg. 3)  The participants also reported that they could recognize a knowledge gap in many scenarios, and unknown medical terminology about Ovarian Cancer was reported as a common trigger of active information seeking, as P08 said: “When I got the original test results on MyChart, there was a bunch of words that I wasn’t really sure what it meant. So, I looked up the words and it said that it was an ovarian tumour.” (Patient. Pg. 3) | **X** |  |  | 9. Patient |
|  | **Active information seeking:**  Seeking Ovarian Cancer survivor stories | Several participants reported that they sought and read stories from other Ovarian Cancer survivors, especially from online social groups, to get knowledge, hope and inspiration. P12 learned about treatment information from survivor stories:  “Reading people’s stories was truly important to me because you could see that their doctor may have taken a different approach. It created questions for me, which was good, that I could go back and question Dr XX about.” (Patient. Pg. 3)  P18 told us how she sought survivor stories from peers on Facebook and benefited from them: “When somebody tells you their story, you can pick up their strength and maybe improve your own strengths. It’s a tough fight.” (Patient. Pg. 3) | **X** |  |  | 10. Patient |
|  | **Active information seeking:** To reduce anxiety and uncertainties | Participants identified information seeking as a way to reduce anxiety and uncertainties and set their minds at ease. For example, P11 said that she sought information to reduce her anxiety associated with cancer recurrence: “for me, right now the anxiety is tied to preventing cancer from recurring. So, the knowledge of what to do is kind of freeing to reduce the anxiety. So I need the knowledge and the confidence that I know what to do.” (Patient. Pg. 3)  P04 is a family caregiver for her sister, and she told us that her sister looked up information to deal with uncertainty associated with Ovarian Cancer: “Obviously, ovarian cancers are a hard one because there are some people who live 15, 20 years with ovarian cancer or get cured, and there’s others that aren’t that fortunate. So I think one of the things with ovarian cancer is dealing with that uncertainty. My sister wants to have hope, but she also wants to be realistic in planning for her children’s future and understanding if there’s—maybe when the right time to seek palliative care is versus putting her body through something that is going to be so detrimental to her quality of life and probably won’t extend it.” (Patient. Pg. 3) | **X** |  |  | 11. Patient |
|  | **Active information seeking:** Being unsatisfied with communication with providers | Some participants shared perceptions that their healthcare providers did not provide sufficient transparency regarding treatment information, leading them to actively seek out information on their own. For example, P05 reported that “It’s like everybody’s in the dark. The doctors don’t tell you what they’re going to give you in advance, and we’ll worry about everything later. I don’t believe that should be the way it should be.” (Patient. Pg. 3)  She also explained why she thought it was important to seek and learn information proactively instead of being passive about clinical trials “when it comes to clinical trials and you building up exclusion conditions, that you may not be able to get into clinical trials because you’ve got the wrong treatment or you chose a treatment that you shouldn’t have if you definitely knew that it would exclude you from a clinical trial.” (Patient. Pg. 3) | **X** |  |  | 12. Patient |
|  | **Active information seeking:** To gain confidence | Participants said that collecting as much information as possible and getting involved in the treatment and decision- making process would help them feel more confident of the treatment they received. P14 said: “I’m not ignorant of the fact that it [reoccurrence] can pop up in some other form. But I got much confidence to keep everything in my hand.” (Patient. Pg. 3)  P03 even said that she felt the nurse who told her not to look up information on Google was “patronizing and insulting”: “one nurse said to me, “Don’t go on Dr Google,” and I thought that was patronizing because I’m like, “I am definitely going to be trying to research and understand this disease.” I didn’t think that was helpful. I thought that was a little bit insulting.” (Patient. Pg. 4) | **X** |  |  | 13. Patient |
|  | **Passive information acquisition:**  Having sustained information resources | Participants reported using routine information resources to passively receive Ovarian Cancer-related information regularly. For example, P5 said: “I have a couple of websites that will kind of give me a daily email update saying, ‘Okay, this is what just came down the pipe.’” (Patient. Pg. 4)  Besides, participants also told us that they occasionally attended the classes or seminars provided by hospitals and encountered information on a range of topics. P17 shared: “UPMC used to have a Survivor series, and they would have seminars or classes or meetings about certain things, subject lines...That’s pretty much been my main sort of knowledge or information. And it could range from—chemo-brain, to nutrition, to anxiety and the mental—from a psychologist or psychiatrist.” (Patient. Pg. 4) | **X** |  |  | 14. Patient |
|  | **Passive information acquisition:**  Having personal connections with health professionals | The participants asked professionals for help to seek information for them or asked for referral in gynaecological cancers. For example, when P11 talked about her primary information source, she said:  “I relied on my brother-in-law, a general doctor, and he researched the top hospital for cancer then the gynaecology department and then found the chairman of the gynaecology department.” (Patient. Pg. 4) |  | **X** |  | 15. Patient |
|  | **Passive information acquisition:**  Having personal connections with cancer survivors | People who have had cancer in the patients’ personal network is another supportive information resource that allows our participants to be passively exposed to Ovarian Cancer-related information. For example, P14 commented: “I have a couple of good friends that have been cancer survivors and gone through it that have been good mentors for me.” (Patient. Pg. 4) |  | **X** |  | 16. Patient |
|  | **Passive information acquisition:**  Being satisfied with communication with providers | They felt satisfied and eased when the providers explained information and decisions to them face-to-face patiently and respectfully. P16 said:  “He’ll [doctor] sit down and explain everything, and if I don’t understand it, he’ll go back over it, and they may give me a paper to tell me what the side effects of the chemo is and everything. And the doctor asks for my opinion, what I think, and he is wonderful.” (Patient. Pg. 4) |  | **X** |  | 17. Patient |
|  | **Passive information acquisition:**  Having trust in providers’ knowledge | Participants who trusted their providers and believed that “they know better than anybody” would tend to be passive information recipients and more likely to rely on providers to tell them what to do or what not to do. P17 said: “That would be my point of preference”. (Patient. Pg. 4) |  | **x** |  | 18. Patient |
|  | **Information avoidance:**  Lacking knowledge to understand the information | The participants reported that they may choose to consciously ignore the knowledge gap and avoid information if they felt overwhelmed by the information. For example, P13 commented: “Just layman terms are great with me. If it gets too complicated, then I get frustrated, and then I get more confused, and then I have to look up more words. And by the time I’m done, I’m getting totally upset.” (Patient. Pg. 5)  Insufficient search expertise could also impede participants from seeking information themselves especially from some online sources, such as digital databases. For example, P05 said she avoided using Cochrane Library, because “it’s so difficult to search in. I tried to go on there, and I have no clue how their search works. It just shows me that they have a million and a half studies.” (Patient. Pg. 5) | x |  |  | 19. Patient |
|  | **Information avoidance:** Unable to evaluate the relevance and quality of the information | The participants also reported a large amount of irrelevant information, which further contributed to their aversion to seeking information. For example, P17 said:  “I’m a little leery about reading things—it’s hard to tell sometimes what’s drug-pushed articles and what’s actual medical exam articles. And I get nervous about not knowing whether or not this relates to my specific process in my journey.” (Patient. Pg. 5)  Also, P11 said, “what I initially read online was really frightening and wasn’t necessarily true.” (Patient. Pg. 5) | x |  |  | 20. Patient |
|  | **Information avoidance:** Fear of exposure to negative information and emotion | Some participants reported that exposure to negative information and emotions could also push them away from an information source permanently. P17 retreated from online social groups and commented: “it would actually make me more sad to be there at times because we were constantly getting new people, which was great, but it was constant reminders of the negative where I’m trying to just move forward with the positive and not have to be reminded every single time I’m there. And so, I did kind of move away from the group and gave myself some space.” (Patient. Pg. 5)  P15 also blamed the online community: “they always sound worse than what I had felt. I have not been—I mean, I’ve been sick, but I have not been like what I’ve heard.” (Patient. Pg. 5) | x |  |  | 21. Patient |
|  | **Information avoidance:** Feeling powerless about cancer curability | P13 told us about her overwhelming feelings toward the information from the American Cancer Society, an authoritative cancer source:  “Everything’s negative there. It says you have a very low chance of living. Ovarian cancer is one of the worst. They give you absolutely no hope. It scares me. That site is one of the main reasons why I stopped looking stuff up.” Participants who believed that they had no control over their disease and were uncertain about the cancer curability chose to avoid information. (Patient. Pg. 5)  P15 told us “I guess there’s the test that basically tells them [doctors], in the blood test, about the amount of cancer maybe in you... I’m trying not to be angry, blame people, or blame anything. Maybe I’m one that puts my head in the sand... I quit reading the results of my tests.” (Patient. Pg. 5) | x |  |  | 22. Patient |
| **Dumas et al. 2021** | Multifactorial Decision-Making | Participants frequently felt overwhelmed by the volume of information shared at diagnosis and memories around diagnosis were often unclear. Shock at the diagnosis, particularly for patients who had enjoyed relative health throughout their lives, was apparent and contributed to feeling overwhelmed.  “When you’re first diagnosed, it’s such a shock to you, you don’t absorb everything that’s being said.” (Patient, Pg. 6)  “I sort of went into zombie mode, it never occurred to me to say no.” (Patient, Pg. 6) | x |  |  | 23. Patient |
|  | Burden of Logistical Issues | Generally, participants felt they had excellent care and support from the cancer centre and expressed sincere gratitude. “It’s like a blanket around you isn’t it.” (Patient, Pg. 7)  Participants had varied access to informal charitable support, largely due to geographical barriers. “It’s just these things on the ground that you have to do as a patient when you’re feeling exhausted.” (Patient, Pg. 7) | x |  |  | 24. Patient |
|  | Side-Effects | Participants described fatigue as the most challenging aspect of their treatment experience, able to manage only the most basic activities of daily living. “I was incredibly weak and then you still have to do things, and you can’t manage it.” (Patient, Pg. 7)  “It’s a matter of dragging my body around to keep up with essentials.” (Patient, Pg. 7) | x |  |  | 25. Patient |
| **Galica et al. 2020** | Healthcare provider support | Many women relied on physical assessments and/or diagnostic tests (e.g., genetic testing or CA-125 [P1, P3, P6, P9, P10, P11, P12]) organized by their oncology healthcare providers (HCPs). These assessments and tests provided women with “reassurance that everything is fine” (Patient, Pg. 4)  “Wandering around not knowing” (Patient, Pg. 4) | x |  |  | 26. Patient |
|  | Knowing, trusting, and prioritizing self | The cancer journey was a transformative experience (P2, P3, P4, P7, P8) that left one woman feeling like she was a “better person” (Patient, Pg. 4)  This introspective process sometimes began with prompts from other people for women to ex amine their own attributes as resources for coping with FCR (P2, P8, P9), where one woman hadn't looked at herself that way (P2) and another thought that thinking about her own attributes for coping with FCR “gives you strength in and of itself” (Patient, Pg. 4) | x |  |  | 27. Patient |
|  | Finding what works | Sometimes the resources women used followed a trial and error approach to find an appropriate resource to suit the women's need. One woman acknowledged “it is getting the right kind of help” (Patient, Pg. 4)  “I guess I just talk about it … with my husband or something. Or, I would seek medical advice from my family doctor. … I will pray about it and think openly. I don't sit back and not do anything about it” (Patient, Pg. 4) | x |  |  | 28. Patient |
|  | Uniqueness and belonging | The most frequently identified group was women's families who were even cited as being the most useful resource for women to cope with FCR: “The support from my family has been very, very useful. … They were positive, gave me the courage to fight it” (Patient, Pg. 4)  Other women identified an appreciation for relationships with other cancer survivors “who get it” (Patient, Pg. 4) | x |  |  | 29. Patient |
|  | Redirecting thoughts and actions | One woman described her self-talk as “[I] just say ‘don't let this control your life. You're in control. You still have choices'. …So, you talk to yourself and make sure that you're not going to go down that black hole.” (Patient, Pg. 5)  Three other women described a 2-step process to coping with FCR (P6, P11, P12) whereby after acknowledging their fear, they moved onto actions to help them cope with FCR: “I thought, ‘This possibility is there’. And, I think it was just simply acknowledging it…. And, if more than that is needed then I know I need to phone either a friend to talk about it [or] contact the family doctor, or the gynaecology oncology team” (Patient, Pg. 5) | x |  |  | 30. Patient |
|  | Preparing for the future | Women described how the focus of their attention changed over the cancer trajectory and how this impacted their FCR. One woman out lined her cancer journey as “chunks of time that you have to work through” (Patient, Pg. 5)  Another woman described the differences in her focus while on, versus off, treatment: “before, you're always preparing for the next treatment or your next clinic appointment. Now you're … done. So now you have more time for that fear factor … now there isn't anything forward” (Patient, Pg. 5) | x |  |  | 31. Patient |
| **Han et al. 2021** | **Prognostic uncertainty as a cause of fear of cancer recurrence. -**  The fear of possibility | One participant (Patient 15), for example, traced the fear she felt at the time of her routine follow-up visits both to the inability to know whether or not her illness “was going to end badly,” and to an irresistible urge to imagine the worst: “every time I come here I’m panicked, just because it is scary to think that I could come back at any time and have a re-occurrence.” (Patient, Pg. 4)  Other participants exhibited the same pessimistic bias in describing their cancer as a “hiding,” “sneaky” threat lurking “somewhere else, just waiting” to return and spread. (Patient, Pg. 4) | x |  |  | 32. Patient |
|  | **Prognostic uncertainty as a cause of fear of cancer recurrence. -**  Restricting possibility: the need for certainty | One participant (Patient 12) argued that reducing her prognosis to a finite “time bracket” gave her a “sense of time” that lessened her worry. (Patient, Pg. 4)  I don’t believe the number absolutely. But it gives me a time bracket. So, I shan’t be worried about things that are going on that I can’t explain, because they’re either old age or cancer. (Patient, Pg. 4) | x |  |  | 33. Patient |
|  | **Prognostic uncertainty as a cause of fear of cancer recurrence. -**  Unrestrictable possibility | Participants argued that medical experts “don’t really know” the prognosis of individuals (Patient 2), that statistics “are not about people” because they simply “mix together” individual lives (Patient, Pg. 4)  This recognition was a source of distress for many participants; Patient 20, for example, reported that even a small “0.5%” probability of a bad outcome was enough to “drive me crazy forever.” (Patient, Pg. 4) | x |  |  | 34. Patient |
|  | **Prognostic uncertainty as an effect of fear of cancer recurrence. -**  The hope of possibility | Participants’ comments further suggested that this association may have originated from a positive bias in the perception and interpretation of uncertainty: a selective, optimistic focus on best-case possibilities, which might be termed “optimizing”—in contrast to the “catastrophizing” that resulted in the fear of possibility. (Patient, Pg. 4)  One participant (Patient 5) viewed even the smallest possibility of a best-case outcome as a reason for hope: “even if there was a 1% chance, I was like it didn’t matter,” (Patient, Pg. 4) | x |  |  | 35. Patient |
|  | **Prognostic uncertainty as an effect of fear of cancer recurrence.**  Expanding possibilities: the need for uncertainty | Patient 2 put it pointedly: “I don’t want to know what stage cancer it is because if I don’t know, it can be anything.” (Patient, Pg. 4)  So maybe that’s why I didn’t want to know originally. I didn’t want any, to know like, “Well this could be really bad,” because I’d be thinking, “I’m not going to survive, you know” (Patient, Pg. 4) | x |  |  | 36. Patient |
|  | **Prognostic uncertainty as an effect of fear of cancer recurrence. -**  Adaptive effects of uncertainty | One participant (Patient 9) viewed maintaining prognostic uncertainty as part of a general strategy “not to borrow trouble”— (Patient, Pg. 4)  One participant (Patient 9) viewed maintaining prognostic uncertainty as part of a general strategy “not to borrow trouble” (Patient, Pg. 4) | x |  |  | 37. Patient |
|  | **Constructing prognostic uncertainty. -**  Highlighting indeterminacy | Another patient stated that she purposefully avoided seeking prognostic information because “Whatever it [prognostic estimate] says, I want to be the outlier” (Patient, Pg. 5)  Participants recounted how their physicians used language supporting the personal inapplicability of prognostic information—e.g., “Every patient is different” (Patient, Pg. 5) | x |  |  | 38. Patient |
|  | **Constructing prognostic uncertainty. -**  Highlighting ambiguity | Study participants highlighted the ambiguity of prognostic information in several ways, including acknowledging the imprecision or unreliability of prognostic estimates for particular patient subgroups, due to shortcomings in empirical evidence— e.g., “research hasn’t been done” (Patient, Pg. 5)  Other participants noted how prognostic knowledge is unstable and “changing at every moment” | x |  |  | 39. Patient |
|  | **Maintaining prognostic uncertainty -**  Relinquishing knowledge | Patient 2 argued that simply knowing she had OC was enough, and that her priority was simply “to concentrate on what we can do,” (Patient, Pg. 6)  While Patient 4 disavowed prognostic knowledge because it would not change her “plan of care” (Patient, Pg. 6) | x |  |  | 40. Patient |
|  | **Maintaining prognostic uncertainty. -** Embracing ignorance | Patient 8 embraced ignorance about her prognosis because she “didn’t want any limit” to what was possible; she believed that acknowledging a poor prognosis posed the risk of turning into a self fulfilling prophecy. (Patient, Pg. 6)  Patient 7 evocatively described this same orientation toward ignorance as a capacity for “holding some amount of the spaciousness of the unknown”— (Patient, Pg. 6) | x |  |  | 41. Patient |
| **Harris et al. 2024** | Care Services | Immediately post-surgery: “I didn’t realize how much I wanted to get out” (Patient, Pg. 4)  Some patients felt their body was “letting[them]down” (Patient, Pg. 4) | x |  |  | 42. Patient |
|  | Experiences of a stoma | The shock of needing a stoma: “I never really prepared myself that I would get a stoma” (Patient, Pg. 4)  Some patients found receiving a stoma emotionally difficult whereas others were more accepting, particularly when they understood that the stoma was essential to prolonging their life. “It was the stoma that was mentally and physically like more traumatic than the actual abdominal wound and surgery itself.” (Patient, Pg. 4) | x |  |  | 43. Patient |
|  | Preoperative experience | Attitude towards surgery: “I looked at the surgery as my way out” (Patient, Pg. 4)  Patients on the neoadjuvant pathway looked at surgery as a goal to work towards and “felt blessed” to be well enough to undergo the operation. (Patient, Pg. 4) | x |  |  | 44. Patient |
|  | Impact of Surgery | Impact on functionality: “I haven’t been as active, but I expected as much” (Patient, Pg. 5)  Emotional impacts: "I’ll...have surgery and then bounce back. You don’t” (Patient, Pg. 5) | x |  |  | 45. Patient |
|  | Coping Mechanisms | Formal and informal support: “...it helps you, you know, talking to people who are going through cancer” (Patient, Pg. 5)  Patients also expressed a desire for informal support groups where they could discuss their experiences with other cancer patients. “I think what would have helped would have been perhaps talking to someone else about it but I didn’t really know anyone else.” (Patient, Pg. 5) | x |  |  | 46. Patient |
| **Jelici et al. 2018** | Navigating Uncertainty | When I first noticed my symptoms, I guess I was just a little bit bewildered but I thought very little of it, I actually thought I may have been developing irritable bowel syndrome, because I felt really well only I had to run to the bathroom more often than I ever have before … I just simply thought something might be a little bit funny and I, but I really thought it was not serious. (Patient, Pg. 1)  As I said, having ignored the symptoms myself because they were so vague, putting them down to other things, I’m trying to help other people not to do the same thing as me. (Patient, Pg. 1) | x |  |  | 47. Patient |
|  | Responsiveness in healthcare | I went and saw my GP the next day….I’m guessing at that point too that she knew what it was, because she would have also had my blood test results, so she didn’t talk to me about that, but she said, “You need to go and see a specialist”. (Patient, Pg. 3)  Oh I just said I had felt bloated and my stomach wasn’t feeling right, and she [GP] just gave me an external examination and could feel it and said right I need you to go and have scans done and x-rays done and things like that. So I did that the next day and had to go back and see her that was on a Tuesday and a Thursday and by that time she had made an appointment for me to see a gynaecologist on the Friday. So everything moved very quickly. (Patient, Pg. 3) | x |  |  | 48. Patient |
|  | Relational Communication | The hospital just sent this particular oncologist to see me and I didn’t like him from the word go, because he walked in and said, “Oh, you’re a bit of a mystery” and I said, “Oh, so you can’t find anything wrong with me?” and he said “Oh yes, you’ve got cancer we just don’t know quite where it is yet.” That’s how he told me. (Patient, Pg. 4)  I sort of said something to one of the nurses, you know ‘what’s going on?’ We were supposed to be being prepped for surgery [to remove suspected ovary cyst] … she sort of got one of the doctors to come in to talk to me and I said to them ‘look what is going on?’ and she said ‘oh the surgery has been cancelled, you have cancer’ and walked out the door. (Patient, Pg. 4) | x |  |  | 49. Patient |
|  | Person-centred information | What I found helpful was when I went in with the results of the CT scan there was an oncologist sitting in there and also a nurse telling me of the physical implications of the surgery I was about to have…Well, that was really helpful, and frightening at the same time. (Patient, Pg. 6)  I didn’t even know I had a stoma until day three…[my husband] said, “Yes, he [the oncologist] did say that … you just weren’t absorbing the information”…”. (Patient, Pg. 6) | x |  |  | 50. Patient |
|  | Preparation for living beyond cancer | I did ask about it [physiotherapy] a couple of times because basically I had major muscles cut right through my abdomen region and from having children and stuff you’ll understand what the impact of that can have, like you can’t drive for 6 weeks blah, blah, blah things like that, but after that how do you recuperate? And there wasn’t any of that sort of like ‘Ok well we will get you onto that and they can give some exercises to do for that’, you know there wasn’t any of that, which I was quite shocked about … if you’ve had major surgery how to recover back from that. (Patient, Pg. 7)  [I would have preferred] just a bit more information on menopause, surgical menopause. … because it didn’t even cross my mind, I’m quite aware that when your ovaries are gone, you know that is your menopause well and truly, but at the time I needed to be refreshed about that … it was something that didn’t even enter my mind until afterwards. And I actually asked the gynaecologist, I said to them “are you sure you didn’t leave a swab in there?” I said “because I’ve got these sweats really bad” and he just said “I’ll put you on a tablet” and it was good, it all just disappeared. But if I had have known ahead I could have asked for that straight off. (Patient, Pg. 7) | x |  |  | 51. Patient |
| **Chou et al. 2019** | Feeling Extreme Anxiety and Uneasiness | “I thought my menopause had begun, but my period came back, and it would not stop. I thought it was a common gynaecologic [issue], similar to excessive vaginal discharge” (Patient, Pg. 102)  The doctor said that the tumour appeared to be in the abdomen, and that drugs must be injected into the stomach. However, I do not recall having heard him explain how [they would] do that. . . . I had no idea what the treatment was. I did not understand how the drugs injected into my stomach were supposed to [work]. (Patient, Pg. 102) | x |  |  | 52. Patient |
|  | Experiencing Specific Symptoms | “I felt that the abdominal catheter seemed stuck on something. I was [afraid] that it had gotten displaced.” (Patient, Pg. 102)  “I had to endure [a lot of] pain when the abdominal catheter [was] being tugged and the needles [were] being injected into my body.” (Patient, Pg. 102) | x |  |  | 53. Patient |
|  | Experiencing Unexpected or Severe Physical Issues and Psychological Discomfort | “clumps of hair would fall off whenever I combed my hair. It was just like what I saw on TV.” (Patient, Pg. 102)  “I felt [nauseated] whenever I smelled food. I simply had no appetite when food was placed in front of me.” (Patient, Pg. 102) | x |  |  | 54. Patient |
|  | Feeling Isolated from People | “I was scared of what others might think of me; I would rather stay at home.” (Patient, Pg. 103)  “I could not resist crying whenever I saw people. I just could not talk. Eventually, I began avoiding people.” (Patient, Pg. 103) | x |  |  | 55. Patient |
|  | Learning How to Manage Side Effects | “According to what I found on the Internet, eating ginger can help. So, I would take . . . ginger before I ate. After that, [I could discharge] the gas in my stomach . . . and I could finally eat.” (Patient, Pg. 103)  “Even [though] I had no appetite, I forced myself to eat . . . because I knew that the nutrition was important to me.” (Patient, Pg. 103) | x |  |  | 56. Patient |
|  | Worrying About Families, Illness Progression, and the Future | “I had no idea if I could be cured. I heard that this type of cancer can recur.” (Patient, Pg. 103)  “I was concerned about recurrence. I was not hopeful about the future at all.” (Patient, Pg. 103) | x |  |  | 57. Patient |
|  | Receiving Support From Others and Accepting the Illness | “My friend was very considerate. He helped me with everything wherever I went. He always listened to me.” (Patient, Pg. 103)  The social worker told me that love is courage. When I used positive thoughts to replace rage, anger, and guilt, [I felt like] my immune system would be activated. That was a huge encouragement, and I began to encourage myself. (Patient, Pg. 103) | x |  |  | 58. Patient |
| **Lee et al. 2020** | Maintaining hopefulness | Like I said, I have to think positive. So that when the thing (ovarian cancer) happens to us again, we won’t feel so down, won’t feel it so much. At the most, 1 or 2 days only, you know (Patient, Pg. 44)  My markers were going up, she (doctor) advised me to have chemo, I didn’t think there was any other option, so I went ahead (Patient, Pg. 44) | x |  |  | 59. Patient |
|  | Avoidance of information | I like it better when I don’t know. I feel at ease. If I hear other people talking about stage 3 and 4 cancer, it is a reminder. Always thinking about the disease, it will make us unable to do anything, we will become sick (Patient, Pg. 43)  It’s been a long time since I’ve found out, so I don’t want to keep the memory here (about cancer recurrence), let it go peacefully, I don’t want to keep remembering. So, in my heart, I’m free (Patient, Pg. 43) | x |  |  | 60. Patient |
|  | Acceptance of condition | I was very sad when I knew (about the recurrence), I cried. Then I thought to myself, “Woah, the stupid thing likes my body” and then I start laughing. “You like to grow in my body, never mind, I’ll let you grow.” I was alone in the room, I thought by myself, “I accept what I have now.” So whatever I can eat, I eat. If my children go anywhere, I follow, I don’t care (Patient, Pg. 43) |  | **x** |  | 61. Patient |
|  | Seeking spiritual help | Whatever it is God gave it to you your richness, your health. So this is just a test. A test from God see how you are patient with his test, how you accept it. So you have to be patient, because whatever we are patient with will mature us. We are “redha,” we have to think positive. We can’t blame this or that (Patient, Pg. 44)  I’m a Christian, so my life is in god’s hands…I’m in good hands. So I’m at peace, I don’t worry…So God gives that strength. If not, emotionally and spiritually, I really want to give up (Patient, Pg. 44) | x |  |  | 62. Patient |
|  | Coping with costs of treatment | The first chemo in (anonymized)’s private center was already RM4500 [USD 1100]. So I said to the doctor, “I can’t afford it, I want to transfer to the government (hospital).” Now, it’s RM 200 (USD 50), I use the guarantee letter, my child works in the government, so from cost, can cover the cost, no problem (Patient, Pg. 44)  My husband is a government retiree. I’m not. That’s why I said I’m not burdened (by cost). Because if we do a CT scan, doctor already writes you have to pay a few hundred, but I don’t have to. Because of my husband, we are not charged. Maybe if my husband wasn’t in the government, self‑employed, maybe I would be worried about being charged for this and that, and many surgeries (Patient, Pg. 44) | x |  |  | 63. Patient |
| **Liang et al. 2022** | Construct: Job demands | One patient recalled trying to return to work but “every time I go back to work or try to go back full time, the cancer returns... So, no, I don’t know if I’ll ever get to go back to work.” (Patient, Pg. 7)  “We don’t have anywhere to put you due to your circumstance. You really need to fill out these papers and retire out” (Patient, Pg. 7) | x |  |  | 64. Patient |
|  | Construct: Cancer demands | I experienced a lot of fatigueless… getting around was hard... socialization changed, I didn’t socialize as much with people.” Another patient explained “when the… effects of the chemo had me so down where I couldn’t get out of bed, [my husband] had to stay home.” (Patient, Pg. 7)  “I hate having to have somebody… wait nine hours… it took my independence away from me.” Another patient stated that she usually is the one helping her family and “it’s humiliating…being on this side now. (Patient, Pg. 7) | x |  |  | 65. Patient |
|  | Construct: Workplace resources and strategies | “[her] a purpose to get up… motivated and going… [so she does not] feel sorry for [herself].” (Patient, Pg. 7)  “to coordinate as many appointments on one day as possible [and tries] to coordinate [her husband’s] appointments with [hers]. (Patient, Pg. 7) | x |  |  | 65. Patient |
| **Matsui et al. 2024** | Confronting the reality of losing their ovaries and uterus | I told the doctor to preserve the uterus if possible if the tumour was benign. The physician asked why, and I said that my body has it because it needs it. It might not be a problem even if it were taken away, but I think that it is in my body precisely because it is necessary. Moreover, I never had any problems with my uterus, so I don’t think it is necessary to remove it (Patient, pg. 5)  It started with suspected cancer, and then I got on the operating table and lost consciousness. I woke up, and then I was told by the doctor that it was cancer … My uterus and ovaries had already all been removed … I just had to accept it, and it just happened without me having any time to think … I couldn’t organize my feelings (Patient, pg. 5) | x |  |  | 66. Patient |
|  | Contemplating the reversibility and irreversibility of womanhood | For the time being, I thought that it was the only time I could do this, so I had fun arranging my wigs and taking pictures. I think I faced the adversity by having fun (Patient, pg. 5)  Up until now, I have lived without actively wanting children. However, there is a profound distinction between being able to have children and not being able to have children. There is no change in the current circumstances, but no matter what I say, I wonder if my words will be dismissed as sour grapes. … I felt a sense of loss for the first time at that point (Patient, pg. 5). | x |  |  | 67. Patient |
|  | Grappling with altered and often negative feelings toward sexual activity | My partner is still in his 40s, but he can no longer engage in sexual activity, and I feel sorry for him and a great sense of responsibility. I think that I should do something, but even that has become tiring (Patient, pg. 6).  It really has just become a painful time … I feel like I’ve become a sex doll … I often get turned off during sex, and I think, What am I doing? … But I do think that this is my role as a wife (Patient, pg. 6). | x |  |  | 68. Patient |
|  | reassessing the essence of partnership | I’m not really good at it; neither of us can say I love you in words directly. So, for example, I would try to express family ties by saying things like, ‘My son and dad are both important family members.’… It’s the same with my husband. My husband would say to our son, such that I could hear him, ‘Mom is sick, so the two of us men should support and protect her’ (Patient, pg. 6).  I think that a marital relationship can be established even without sex. Sometimes, I feel like hugging, so I started to express affection in this way. Past physical expressions of affection were sexual activities and kisses, but… if you ask me if it’s fine like this, I would answer that’s not the case, and I’d like to try again, but there’s no opportunity for that (Patient, pg. 6). | x |  |  | 69. Patient |
|  | Finding contentment in their identity as women | When my menstruation resumed, I thought to myself, ‘Oh, it’s back to normal.’ I felt a strange sense of accomplishment, as if the bad parts were completely gone, leaving the essential parts intact. I remember that feeling very well. That it had finally come back. I think it was about a year after the treatment ended. That my body had recovered (Patient, pg. 6).  Before I was sick, I was not pursuing femininity. I think that was probably an inferiority complex. I really wanted to reject femininity and dismiss it. I wanted to reject the notion that women should not be assertive. But that’s not because they are women. I felt that if I can’t do it, I should live life the way I want (Patient, pg. 6). |  | **x** |  | 70. Patient |
| **Moskalewicz et al. 2022** | Regret and guilt | “I wonder whether I could have changed anything, whether I could have done something differently.” (Patients, pg.4) | x |  |  | 71. Patient |
|  | Distant happy past | “I like flashbacks (…) Things come back, you look at something, a souvenir from a friend (…) and memories come back “ (Patients, pg.4) | x |  |  | 72. Patient |
|  | Living in the now | “Throughout this disease, I have lived from day to day” (Patients, pg.4) | x |  |  | 73. Patient |
|  | Explicit passing of time | “I have my cell phone and I look at the time, very often “ (Patients, pg.4) | x |  |  | 74. Patient |
|  | Chemo-clock | “I live from appointment to appointment “ (Patients, pg.4) | x |  |  | 75. Patient |
|  | Paradox of time | “I am always looking at my watch, when will the day pass? To feel better (…) And then after this Friday, this first week, I am like, oh God, let it fly slowly, so that I don’t’ have to go. “ (Patients, pg.4) | x |  |  | 76. Patient |
|  | Short temporal horizon | “We had plans, always some, not especially far reaching, but for two, three months, up to a year (…) At the moment I don’t plan anything. I don’t have plans” (Patients, pg.4) | x |  |  | 77. Patient |
|  | Losing and gaining control over time | “I would like the nights to pass quicker, when I don’t sleep, the nights are so much dragging, when one’s in pain” (Patients, pg.4) | x |  |  | 78. Patient |
|  | Unpredictability | “It is at the back of one’s mind, this disease is there, and one doesn’t know when it can attack (…) I have this awareness that something might happen” (Patients, pg.4) | x |  |  | 79. Patient |
|  | Finitude and death | “I always previously thought that I have time. If not now, then later (…) Now, it is not like that anymore” (Patients, pg.4) | x |  |  | 80. Patient |
| **Newell et al. 2025** | HRQoL | It's just so taxing mentally, too, because you're so tired. All you want to do is lay down and go to sleep, and you lay down, and you sit there with your eyes open until you fall asleep, and then you finally get to sleep, and you wake up and it's like 5 min later. You're like, “Oh, my God! Why am I awake?!” (Patients, pg.45)  “It's extremely hard, especially for someone who is so active. I made up a story for my hiking buddy. I said I was doing something else, and I wasn't. I just couldn't get up.. I knew I wouldn't be good company for going for a… I was right here on the couch with the remote and taking naps because I was so out of it.” (Patients, pg.45) | x |  |  | 81. Patient |
|  | Sleep experiences | “They put me on the 2 maintenance drugs… At first I was on 300 mg of [PARP inhibitor], and I couldn't sleep. And I was exhausted, but I couldn't sleep. I'd wake up for 3 h during the middle of the night and read and then sleep for 3-h increments.” (Patients, pg.45)  “I was really, really struggling with sleep a lot. Then, actually, when I started chemo for the recurrence, I think the chemo kind of reset my system. And now I'm actually able to sleep better. Which has been really great, because I was pretty miserable.” (Patients, pg.45) | x |  |  | 82. Patient |
|  | Fatigue experience | “Originally, when I was on [chemotherapy], I would go on Friday and get my treatments, and by Monday or Tuesday. I was just wiped out for like a day or two… But then it would slowly wear off, and I'd get more energy, and then I go in again, so that had its own sort of cyclical thing.” (Patients, pg.45)  “I had more energy when I was going through chemo… and even after the surgery, I was up and walking 2 days after the surgery… But it's the [VEGF inhibitor] and the [PARP inhibitor] that have created the fatigue that have created the weakness… The biggest side effect would be fatigue. I've never had fatigue like that before.”(Patients, pg.45) | x |  |  | 83. Patient |
| **Pozzar et al. 2019** | Patient–Provider Relationship | It began to feel . . . like an assembly line. Like, come in, number 1,024. Mark her off. Reminding me that I’m gonna die, just in case I didn’t catch it. I said, “If I go to another hospital, they may not be as well renowned, but maybe they will listen to me and maybe they’ll fight for me.” Because [at this hospital] . . . I feel like no one is fighting for me. They’re just waiting for me to die. (Patients, pg.598)  I was really kind of appalled by how blunt [the surgeon] was. But I did get a chance to talk to one person he operated on. Four years later, [she] is still alive with no evidence of disease. I respect that his strengths are elsewhere. (Patients, pg.598) | x |  |  | 84. Patient |
|  | Support | [My friends] never really reached out to me . . . so I reached out to [the hospital]. But they didn’t really have a support group for post-chemo patients. I just decided, “You know what, I’m gonna just deal with this myself.” I weeded out a lot of people. (Patients, pg.598). | x |  |  | 85. Patient |
|  | Self-Concept | I ran my first half marathon with the tumor inside me. I was 32, 33. I didn’t know how [chemotherapy] would affect my body. I was excited to get back into running afterwards, so I didn’t want to put that strain on [my body]. (Patients, pg.598).  “I wanted to keep it cool for my kids, and I think by going back to work as soon as I could, that kept my kids in a good frame [of mind]” (Patients, pg.598). | x |  |  | 86. Patient |
| **Roche et al. 2016** | Barriers to Care and the “Little Big Things” | So, care wise...I really am very grateful…systems wise [chuckles], that’s where I had my problem. (Patients, pg.977).  The big things seem to work okay. It’s...all these little things…and they’re not really little. (Patients, pg.977). | x |  |  | 87. Patient |
|  | Consistency and the Human Element | I just feel like there needs to be somebody who manages, who helps you manage all the administrative pieces, all... those kinds of things...that you can depend on to get the right information. (Patients, pg.977).  I found communications were very confusing at first. Who to talk to, you know, to call anytime. You know, “this is the number,” and then you might phone that number, you might make it a callback, and then you might get someone you don’t recognize. (Patients, pg.977). | x |  |  | 88. Patient |
|  | Timing | Nil provided |  |  | **x** | 89. Patient |
|  | Making Change | They should give you an idea on paper who is your exact team, who you contact for different things. [Having a care team] also allows when you do go and see the doctor, to focus on the things that need to be focused on. (Patients, pg.977). |  | **x** |  | 90. Patient |
| **Smith et al. 2024** | Theme 1. Patients are concerned about the financial toxicity of cancer treatments, even when they do not experience financial toxicity themselves | “ Having cancer is very expensive. My insurance plan which is supposed to be like pretty good is ridiculously expensive [before employer contributions].” (Patients, pg.172).  “Oh, I always tell my grown children we would be living in their basement if we didn't have the TRICARE for life.” (Patients, pg.172). |  | **x** |  | 91. Patient |
|  | Theme 2. Patients prioritized treatment efficacy over costs of care | my mind that's not the doctor's issues to worry about what it is costing a patient. And I think that we would have paid anything, I guess. But we're not gazillionaires” (Patients, pg.172).  I think it's my job [to deal] with the insurance company. I don't want them to change the prescription … I mean, I would have preferred somebody else pay for it, but I just, I'm a problem solver. So I do what needs to get done to make what I want to happen, so.” (Patients, pg.172). | x |  |  | 92. Patient |
|  | Theme 3. Clear communication and rapid dose reduction helped patients manage the physical and mental side effects of PARP inhibitor maintenance | “It's like more like, research studies have focused around that three-year timeline. But everybody's different and it depends on how you react…” (Patients, pg.172).  “And so we're kind of in this routine where I just get my labs done and then talk to the nurse practitioner pretty regularly and as long as my labs are stable, there's not a lot to talk about.” (Patients, pg.172). | x |  |  | 93. Patient |
|  | Theme 4. Patients appreciated the chemotherapy break afforded by PARP inhibitor maintenance therapy, even if their cancer recurred | “… because that's my thing, [I'm a] walker, decades long thing. So I kept that up and with this [PARP-I] I just I was like, wow, this is the answer. I'm on this PARP Inhibitor and my hair is coming back and I feel great.” (Patients, pg.173).  “I mean, I had a lot of that, a lot of problems with chemotherapy. So I was hoping and it was true that this has much less side effects and enables me to do more.” (Patients, pg.173). |  | **x** |  | 94. Patient |
|  | Theme 5. Home delivery facilitated patient adherence to PARP inhibitor therapy | “Yeah, they usually call me and coordinate delivery date and then it shows up on my doorstep. So it doesn't get much easier than that.” (Patients, pg.173).  “It wasn't a process. It just came.” (Patients, pg.173). | x |  |  | 95. Patient |
|  | Theme 6. Transportation is a barrier to cancer care, especially in older patients. | “While they give you valet parking, free parking on actual transfusion days, but when you go for[other] things…checkups or whatever, there's no free parking.” (Patients, pg.173).  “It never dawned on me, parking transportation, all the things that are to get you there just to get the treatment and the things that you need.” (Patients, pg.173). | x |  |  | 96. Patient |
|  | Theme 7. Patients understood the role of germline and somatic testing in their use of PARP inhibitors. | “I'm pretty sure that was the driver. And in fact, what the remember the oncologist telling me my doctor after the diagnosis came out, I mean that I was BRCA positive. She said that's actually good news because the outcomes are better for BRCA positive because of PARP inhibitors, so.” (Patients, pg.173).  “I don't know that I would have even been on the PARP inhibitor. I think we would have just treated it with regular chemo, and I would have moved on. But since I had the genetic mutation that's where she felt that we needed to go the extra step and start looking at some other treatments that were out there.” (Patients, pg.173). | x |  |  | 97. Patient |
|  | Theme 8. Patients perceived telemedicine and clinical trials as improving access to, and reducing costs of, care. | “It just interfered a lot less with my life. I didn't have to take the day to go to the doctor. And there's no waiting.” (Patients, pg.174).  “It's never the same on a video call” (Patients, pg.174). | x |  |  | 98. Patient |
| **Staneva et al. 2019** | Optimistic Tenacity: ’’You Just Have to Be Strong and Keep Going’’ | Donna: the first doctor that I saw-the very first day he called me a stoic woman. Because I didn’t cry or anything like that, you know, I just-you just have to accept it. Interviewer: And did you appreciate being called a stoic woman? Donna: Yes, yes. Definitely. Interviewer: And you decided to-to keep up with that definition? Donna: Yes, yes as much as I could, yes. Interviewer: So in a way that was helpful? Donna: Yes definitely!’’ (Patients, pg.35).  Oh I just pulled myself together and just um-you just have to get on with it like, um, some nights I would, um-if I couldn’t sleep I would just have a bit of a cry by myself, and I think that sort of relieves the tension. No I didn’t seek emotional support. I felt as if I didn’t need it because as I said to you he [the doctor] called me a stoic woman. (Patients, pg.35). | x |  |  | 99. Patient |
|  | Self-care: Nourishing My Body, While Poisoning My Cancer | Because of the urgency of it, you don’t have time to think of alternatives [I] we don’t have a lot of options in Australia; I think, ah, um, knowledge should be universal not confined to a country or whatever (yeah), um, and I think there should be more investigation into alternatives (yeah), um, I really do. (Patients, pg.35).  I did a lot of alternative treatment as well; that involved other days during the week to help protect my organism on the inside against the effects of the drugs of the chemotherapy. [I] I believe that the natural therapy that I was taking is what helped me and my body cope with chemo. (Patients, pg.35). | x |  |  | 100. Patient |
|  | Support Systems: ’’Worth Their Weight in Gold’’ | Everybody was wonderful [I] angels! They treated you really nicely. They’ve got so many patients to deal with, and yet they can spend time with you, and they’re just so nice. (Patients, pg.36).  It might sound a bit weird. I think the fact that they were so matter of fact about it like it wasn’t a lot of sympathy, it wasn’t a lot of bull….it was just kind of like, oh yeah, you know, and, um, I appreciated that. (Patients, pg.36). | x |  |  | 100. Patient |
| **Stilos et al. 2018** | Confronting the Initial Diagnosis | I came to my own conclusion about what stage 4 meant. You know nobody really explained what we (might) need? What to expect? What’s coming next? I feel like we got none of that information at alI’’ (Family, pg.466).  ‘‘We were both desperate to know I was constantly going to the Web site and seeing, OK now this is happening, now does that mean it is in a worse stage? Like how much time do you have when you are in stage 4I’’ (Family, pg.466). | x |  |  | 101. Caregiver |
|  | Dealing With Fluctuations in Physical Status | ‘‘I when my mom was getting sicker, I was kind of waiting for them to say ‘I how are you managing at home?’ I And I felt I often had to take the ball in my own hands isn’t there any seat we can put on the toilet a shower chair a bedrail to help my mom?’’ (Family, pg.466).  ‘you can’t lose hope, there’s always hope and try not to let people take it away I wouldn’t say I was always like that every day for 3 years, but I was, I had to be on some level hopefully I always found something to be happy about or hopeful about’’ ((Family, pg.466). | x |  |  | 102. Caregiver |
|  | Confronting the Turning Point | ‘A week or 10 days before she died, my sister and I were there she was definitely going downhill she was having all her usual issues, but there was no reason for us to believe [death was near] until I got the call from my dad’’ (Family, pg.466).  ‘‘I phoned my older brother that weekend because he was supposed to come in 2 weeks’ I said ‘no she’s got about a week.’ And I said, ‘get a flight tomorrow,’ so he did’’ (Family, pg.466). | x |  |  | 103. Caregiver |
|  | Facing Decline and Death | ‘‘[the doctor said] you can go into palliative care, and my mother was leaning in that direction, but my father really didn’t want it cause he was worried they would start shutting the family out’’ (Family, pg.466).  ‘if I was able to read how advanced ovarian cancer fared or what their experiences were I think that would have prepared me for what I thought she could have been going through to relate, and I might have been able to find that somewhere on the Net, but nobody was offering it, that information directly to us’’ (Family, pg.466). | x |  |  | 104. Caregiver |
| **Tan et al. 2020** | Informational needs | it was my lack of knowledge of the signs and symptoms of ovarian cancer… ensure as many women as possible realize what these signs and symptoms are and that they need to be very proactive. (Patients, pg.215). |  | **x** |  | 105. Patient |
|  | Healthcare | My original oncologist was a generalist and not at all interested in treating me as an individual. I have learned that choosing the oncologist is so important… (Patients, pg.215). | x |  |  | 106. Patient |
|  | Isolation | I still feel quite isolated. I lost many friends ‐ people just disappeared from my life, I think they couldn't face talking to me about cancer. I wish I just felt grateful to be here, I know I'm lucky to have survived and not everyone is as lucky. (Patients, pg.215).    OC is very isolating as it does not feel as though there are many affected (alive) women out there, especially young women… (Patients, pg.215). | x |  |  | 107. Patient |
|  | Disruption | In a matter of a week after my diagnosis, I didn't look like myself or feel like myself anymore… I was terrified I'd never feel normal again, and worried about how to get on with my life. (Patients, pg.215). | x |  |  | 108. Patient |
|  | Treatment related difficulties | Then the operation, followed by weeks of chemo, and its associated side effects. Although my cancer diagnosis was several years ago, I am still living with those side‐effects… (Patients, pg.215). | x |  |  | 109. Patient |
|  | Access to services | Most groups I went to were full of breast cancer patients and survivors ‐ it is alienating to be the only 'non‐pink' cancer person in the room… 2 years post diagnosis and 4 different support groups and I still have not met a single other person with Ovarian cancer (Patients, pg.215).  My surgery … was to be at a hospital 250km away from my home which meant an expense for accommodation for my partner as well as spending 7 days and nights in a town different from my kids … (Patients, pg.215). | x |  |  | 110. Patient |
|  | Social supports | Lucky for me I have a very supporting husband who is helping me through all this but at times I still feel very much alone. (Patients, pg.215). | x |  |  | 111. Patient |
|  | Response/coping styles | I am so grateful… BUT. The other thing was mental thoughts. Will it come back, how will I be affected, etc… I always push them to the back of my head, sometimes, it just pops up. (Patients, pg.215). | x |  |  | 112. Patient |
|  | Caregiver perspective | It was difficult to hear some of the details, but also prepared me for what was inevitably going to happen… The communication and keeping me informed assisted me in coping. (Patients, pg.216).  The most challenging aspect … is the total helplessness that I felt. I was there to give her support and love, but I couldn't control how she was affected by her treatment. (Patients, pg.216). | x |  |  | 113. Caregiver |
| **Teeteh et al. 2017** | Feeling different | It was one good year after I finished treatment before I started to feel like myself a little bit more. Even when I look in the mirror now, I feel that I am so old-looking; I really feel like it aged me. (Patients, pg.67).  It affected the way I perceive myself. I feel so deformed and so ugly; I hate looking at my body in the mirror. I have no interest in having a relationship with a man. I would love to get married but I do not see it in my future. If I cannot stand to look at myself in the mirror, I cannot even imagine a man would. (Patients, pg.67). | x |  |  | 114. Patient |
|  | Seeing marks | The nurses were there and I said, “did they do a hysterectomy?” and both of them looked at me with this terrified look and said “yes, they did.” I could not stop crying for over an hour. Even though I had someone come in [later] to tell me that it did not happen, it was still like the end of the world for me because I want to have a lot of kids. (Patients, pg.69).  I had 25 staples on my stomach; I came home with drainage tubes because I was still draining. I have scars from my belly all the way down and scars from drainage; so anytime someone makes a motion around their stomach I feel that it is because my stomach is fat. (Patients, pg.69). | x |  |  | 115. Patient |
|  | Moving past the scars | [The support group] helped me gain my self-confidence back because with my chemo, no matter what I did I gained 10-15 pounds; and I just did not look like myself. I had no hair, I had no eye-lashes, I had no eyebrows. (Patients, pg.70).  My brother had a hard time sitting there looking at me with no hair because that made it very real to him. I could see it all over his face; he just kept looking at me and his lips quivered and he had a very hard time because with the hair gone, it made it real to him. (Patients, pg.70). | x |  |  | 116. Patient |
| **Thomas et al 2018** | Survivorship is more about living than surviving | Participant A: “Most concerned about chemo & vomiting.”  Participant B: “Women seem to seek first to know they are not alone and also practical concerns that come with treatment.”  Participant C: “I feel what is missing is more stories of women living with OC. When I was first diagnosed I was sure I would not survive.” |  | **x** |  | 117. Patient |
|  | Recurrence is such a hard topic, but I've read too many stories of women who felt slammed because it wasn't discussed at all | Participant E: “Need clear guidelines [for] long-term remission after multiple recurrences, follow up-scans, CA-125. [Gynaecological oncologist and medical oncologist] opinions differ.”  Participant C: “Leg pain from [carboplatin/Taxol] still present. Issues with my sleep. 6 months post chemo. Told leg pain was a chemo gift and would stay.” |  | **x** |  | 118. Patient |
|  | We still have work to do to improve gynaecology patient education on side effects, emotional needs | Participant B: “Serve ovarian cancer community as research advocate and provide peer support.” |  | **x** |  | 119. Patient |
| **Tsai et al. 2020** | A depressed state | “The third stage of ovarian cancer was identified I collapsed and cried ‘Am I been sentenced to death’.”  “The doctor said I probably don't need chemotherapy after operation. Later, I told need chemotherapy if the index not decreased. The information was contradictory.” (Patients, pg.117). | x |  |  | 120. Patient |
|  | Shadow of cancer recurrence | “My physical strength and memory deteriorated. I felt numbness in my tongue.” She also mentioned her menopause came early after surgery. (Patients, pg.117).  : “In the face of follow-up tests of the index, it feels like a time bomb. Usually, irregular sleep, diet, and excessive fatigue can cause the index to rise.” (Patients, pg.117). | x |  |  | 121. Patient |
|  | A change of mindset to move forward | “I was calculative and strict over details; now, I learn to cherish with a broader view.” (Patients, pg.117).  “Panic and despair stroke me during recurring, surgery, changing chemotherapy drugs. I meditate on the scriptures and repeat praying. The power of faith is beyond imagination.” (Patients, pg.117). | x |  |  | 122. Patient |
| **Webb et al. 2023** | Fear and uncertainty | There’s a lot of fear about what will come though, that’s probably my fear. (caregiver, pg. 7)  I mean if she has to go through another lot of chemo… I don't think that'd be worthwhile, I don't think she would go through chemo again it's just been horrendous, this last lot. (caregiver, pg. 7) | x |  |  | 123. Caregiver |
|  | Hopelessness | …it was hard, watching [person] go through it. And, all I could do is just support. I can't cure her…I found it quite hard to watch someone you've known and loved for 45 years. (caregiver, pg. 7)  If there was going to be help overseas for her, whether we could go that way if needed…We would do whatever it takes to help (caregiver, pg. 7) | x |  |  | 124. Caregiver |
|  | Liminality | I think I just sit back and wait, because there's nothing I can do, except looking after my wife right now. So, we have, a life, the best possible life till the cancer comes back. (caregiver, pg. 7)  I'm in a bit of limbo in between states and in between, knowing what my longer‐term plans are. It's a bit hard to plan that stuff. (caregiver, pg. 7) | x |  |  | 125. Caregiver |
|  | Caregiver's protection of the person and self (role as protector) | My concerns for myself are nothing compared to what your partner’s going through. You know, so you sort of think I’ll just keep a lid on it or keep it all nice and calm and stuff… because of that attitude you don't see any build‐up of emotions and things like that (caregiver, pg. 8)  We sort of try and – try and keep it as positive as possible and always look on the brighter side and look at the bigger picture. (caregiver, pg. 8) | x |  |  | 126. Caregiver |
| **Williams et al. 2025** | Barriers exist that affect clinical trials awareness and participation | “There’s a whole lot of women who are from disadvantaged backgrounds or non-English speaking backgrounds” (Patients, pg.7).  “Barriers for me would be… just the energy to invest in looking for information whilst I’m undergoing active treatment” (Patients, pg.8). | x |  |  | 127. Patient |
|  | Theme 2: Instigating the conversation and doing my own research | “I’m always the one that sort of brings it up and says, is there anything? it’s me instigating that… question” (IP3).  “I started just researching myself, overseas trials. When I come back to my oncologist I've said, ‘What about this trial? What about this test?’” ((Patients, pg.8). | x |  |  | 128. Patient |
|  | Theme 3: Finding solutions to improve clinical trial awareness and information access | “I think that centralised repository… would be the best way to go forward” (Patients, pg.9).  “I did the filtering and there’s 52 options to go through and six pages” (Patients, pg.10). | x |  |  | 129. Patient |
|  | Theme 4: Altruism is a motivator | “I’m gonna do my best for everyone out there, to help” (Patients, pg.9).  “I don’t mind if… it won’t help me, obviously I’d love it to help me, but if it it’s gonna help other people down the track, obviously that’s a bonus too” (Patients, pg.10). | x |  |  | 130. Patient |
|  | Theme 5: Emotions regarding clinical trials are varied | “Ovarian cancer definitely needs money, and I always get a little bit titchy when breast cancer gets so much. I keep saying, what about me? What about ovarian cancer?” (Patients, pg.11).  “I feel very lucky that my surgeon worked with the surgeon running the clinical trial. I don’t know how that’s disseminated otherwise… You know, is that just luck of the draw?” (Patients, pg.11). | x |  |  | 131. Patient |
| **Yan et al. 2022** | Basic Knowledge of Genetic Testing as a Medical Test | NiL |  |  | **x** |  |
|  | Genetic Testing Process | NiL |  |  | **x** |  |
|  | genetic testing implications for patients, | Within the last couple of days there was new information about BRCA women who had ovarian cancer (I think BRCA2 not sure) and new chemotherapy available for that. Has anyone else who has ovarian cancer gone for BRCA testing? If so, what type of chemo did you get? (Patients, pg.43).  [Has] anyone had to undergo a prophylactic mastectomy to PREVENT breast cancer? I have tested positive on genetic testing after stage 3 ovarian cancer and now [doctors are recommending] the mastectomy. Have many questions! (Patients, pg.43). |  | **x** |  | 132. Patient |
|  | implications for family members | When my test returned as positive. I have only one concern. I worry about passing [the genes] to my kids (Patients, pg.44).  [My niece] had 3 children and she’s done having children. Her genetic makeup is kind of similar to ours, and probably that would be something she could have monitored easily and if she did carry that and was concerned, she could have her ovaries removed before she had any problem. I think if you find you are predisposed of having breast cancer, there are somethings you can do to minimize your risk. My sister is correct that knowledge is power. (Patients, pg.44). | x |  |  | 133. Patient |
|  | Terminology | Probably on a website, even on an app. I mean, because you know, it wasn't until I was diagnosed with cancer that I realized there's so many apps out there that talk to other people going through what you're going through...And they post like what they're going through, what kind of meds they're on, what kind of chemo they took. And it kind of makes you understand what other people are going through. And so, it kind of helps you, and then you know if there was something like that too [about GT], and that would help person. (Patients, pg.45).  I would want it printed. Okay. I'm still old school...in spite of designing computer systems for a living. I still like paper. |  | **x** |  | 134. Patient |
